# High-Quality Conformer Generation with CONFORGE: Algorithm and Performance Assessment

**DOI:** 10.1021/acs.jcim.3c00563

**Published:** 2023-08-25

**Authors:** Thomas Seidel, Christian Permann, Oliver Wieder, Stefan M. Kohlbacher, Thierry Langer

**Affiliations:** † Department of Pharmaceutical Sciences, Division of Pharmaceutical Chemistry, University of Vienna, Josef-Holaubek-Platz 2, 1090 Vienna, Austria; ‡ NeGeMac Research Platform, Department of Pharmaceutical Sciences, Division of Pharmaceutical Chemistry, University of Vienna, Josef-Holaubek-Platz 2, 1090 Vienna, Austria; § Christian Doppler Laboratory for Molecular Informatics in the Biosciences, Department of Pharmaceutical Sciences, Division of Pharmaceutical Chemistry, University of Vienna, Josef-Holaubek-Platz 2, 1090 Vienna, Austria

## Abstract

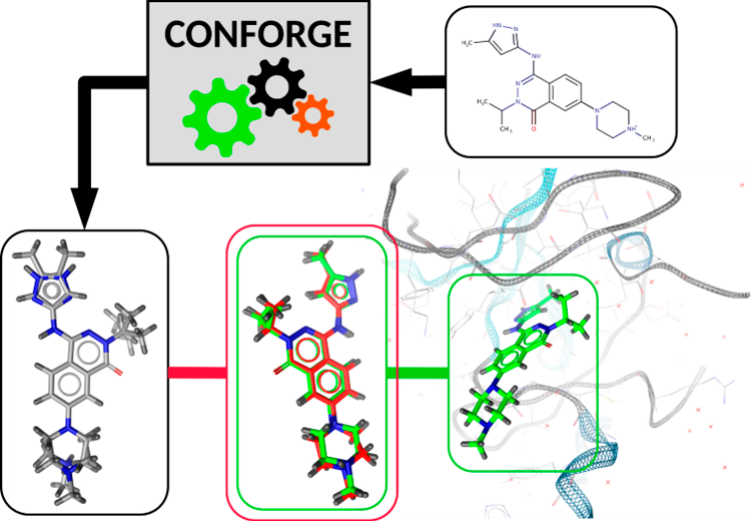

Knowledge of the putative bound-state conformation of a molecule is an essential prerequisite for the successful application of many computer-aided drug design methods that aim to assess or predict its capability to bind to a particular target receptor. An established approach to predict bioactive conformers in the absence of receptor structure information is to sample the low-energy conformational space of the investigated molecules and derive representative conformer ensembles that can be expected to comprise members closely resembling possible bound-state ligand conformations. The high relevance of such conformer generation functionality led to the development of a wide panel of dedicated commercial and open-source software tools throughout the last decades. Several published benchmarking studies have shown that open-source tools usually lag behind their commercial competitors in many key aspects. In this work, we introduce the open-source conformer ensemble generator CONFORGE, which aims at delivering state-of-the-art performance for all types of organic molecules in drug-like chemical space. The ability of CONFORGE and several well-known commercial and open-source conformer ensemble generators to reproduce experimental 3D structures as well as their computational efficiency and robustness has been assessed thoroughly for both typical drug-like molecules and macrocyclic structures. For small molecules, CONFORGE clearly outperformed all other tested open-source conformer generators and performed at least equally well as the evaluated commercial generators in terms of both processing speed and accuracy. In the case of macrocyclic structures, CONFORGE achieved the best average accuracy among all benchmarked generators, with RDKit’s generator coming close in second place.

## Introduction

The vast majority of compounds in the drug-like chemical space comprise one or more rotatable bonds and thus offer some degree of variability regarding their three-dimensional (3D) structure. In solution at room temperature, this structural flexibility is manifested in an equilibrated mixture of multiple interconvertible conformational states that correspond to local energetic minima on the potential energy surface. The bioactive conformation of a drug molecule adopted upon binding to the target receptor is usually quite close to one of its low-energy conformers in solution but can also equal a higher-energy conformational state if increased structural strain is outweighed by a significant gain in energetically favorable binding site interactions.^1^

Computer-aided drug design (CADD) methods that perform an assessment or prediction of the receptor binding capability of molecules rely on the knowledge of the molecules’ bound-state conformation. Unfortunately, experimental data on the bioactive conformations of small molecules are only available for a small fraction of the chemical space. For the majority of molecules—especially those that exist only “virtually”—bioactive conformations have to be predicted as accurately as possible by specialized *in silico* methods. An established approach for computing potential bioactive ligand conformations without requiring prior knowledge of the receptor structure is to sample the low-energy conformational space of the ligand and derive a representative conformer ensemble. In most cases, some of the low-energy conformers in this ensemble can then be expected to closely resemble bound-state ligand conformations. Since the generation of conformer ensembles is a prerequisite for the application of many CADD techniques like structure-based virtual screening (VS),^2^ 3D ligand-based VS,^3,4^ ligand-based pharmacophore modeling, pharmacophore-based VS,^5,6^ and 3D quantitative structure–activity relationship (QSAR) studies, a plethora of dedicated commercial and open-source software tools emerged during the last decades. Well-known commercial tools for the purpose of conformer ensemble generation include iCon,^7^ Omega,^8^ ConfGen,^9^ CAESAR,^10^ Conformator,^11^ COSMOS,^12^ Molecular Operating Environment (MOE) LowModeMD,^13^ MOE Stochastic and MOE Conformation Import,^14^ and ForceGen.^15,16^ Corresponding open-source tools include, e.g., FROG2,^17,18^ Confab,^19^ BCL::Conf,^20^ Balloon DG and GA,^21^ Multiconf-DOCK,^22^ and the conformer generation functionality provided by the cheminformatics toolkit RDKit.^23^ Based on the employed search strategy, applied methods for conformational sampling can be divided into two major categories: (i) systematic and (ii) stochastic approaches.

With the first approach, conformers are generated by the systematic alteration of the torsion angles at the rotatable bonds of the molecule. A benefit of this approach over stochastic sampling is that the number of conformers that must be generated for an exhaustive sampling of the conformational space is exactly defined. Furthermore, the computation of conformers is significantly faster for typical drug-like molecules since multiple conformers can be generated from a single starting 3D structure by just having to perform sequences of simple rigid-body rotations. However, an exhaustive systematic sampling of conformers is usually feasible only for smaller numbers of rotatable bonds due to the combinatorial explosion in the amount of conformers to generate. Nevertheless, the number of rotor bonds that can be handled by this method is still large enough to process most drug-like molecules without any issues.

Stochastic sampling, in contrast, explores the conformational space in a random manner, which results in changes of torsion angles at all rotatable bonds at once. Therefore, this sampling strategy is especially suitable for molecules with high numbers of rotatable bonds or flexible macrocyclic ring systems since representative conformer ensembles can be obtained with relatively few iterations. Software tools implementing a stochastic sampling approach^21,23^ often employ distance geometry (DG)^24,25^ to generate random conformers of the molecule. DG is based on the principle that all possible conformations of a molecule can be described by pairwise atom distance and volume constraints. To this end, lower and upper distance bounds for all pairs of atoms in a molecule are specified in a distance bounds matrix. In addition, a set of tetrahedral volume ranges of four-membered atom groups may be specified in order to enforce the planarity or particular configurations of stereogenic centers. The specified distance and volume constraints then serve as input for an embedding procedure that generates a set of atom 3D coordinates matching the given constraints. A specification of reasonable atom pair distance bounds is crucial to obtain proper 3D output structures. Therefore, empirical information like ideal bond lengths, bond angles, and torsion angles is often used to construct the distance bounds matrix. Nevertheless, the 3D structures generated by the embedding procedure are still quite raw and may contain a considerable number of geometric errors. In order to obtain structurally sound low-energy output conformers, the raw conformers have to undergo an additional structure refinement procedure which can be computationally quite expensive for larger molecules (e.g., iterative force field energy minimization). A further issue with stochastic sampling arises from the fact that it is hard to judge whether the conformational space has been sampled thoroughly enough. Depending on the flexibility of the processed molecule, pursuing the naive approach of sampling a fixed number of unique conformers may then bear the danger of under- or oversampling.

Implementations of both systematic and stochastic sampling often make use of empirical rules and knowledge as well as structural information derived from experimental data like X-ray structures to increase the speed of conformer generation and/or to improve the quality of the output ensembles. Frequently, empirical data are stored in the form of fragment 3D structure/conformer databases, ring conformer templates, or torsion angle libraries, which are then used for fast fragment-based construction of molecule 3D structures or directed exploration of conformational space employing only torsion angles that, e.g., occur predominantly in crystallographic structures. Examples of conformer ensemble generators pursuing a rule-/knowledge-based approach are iCon, Omega, CAESAR, Confab, ConfGen, FROG2, Conformator, COSMOS, and BCL::Conf.

Irrespective of the conformer sampling approach employed, conformer ensemble generators always have to face the challenge of achieving a balance between the conflicting objectives of accuracy (commonly measured as the minimum heavy-atom root-mean-square deviation (RMSD) between the experimentally determined bioactive conformation and any of the conformers in the generated ensemble), ensemble size, and processing time. Of course, the ultimate goal is to generate small ensembles that accurately reproduce receptor-bound ligand conformations with diminishing low computational costs. Since these demands usually cannot be met for all objectives at once, varying emphasis may be put on each parameter depending on the targeted application scenario. If accuracy is of the utmost importance, the choice of a more thorough but also more computationally expensive sampling algorithm may be adequate. If large numbers of molecules have to be processed (e.g., preparation of databases for virtual screening), smaller ensembles may be preferred in order to reduce the storage consumption of the generated conformers and to speedup subsequent processing steps (e.g., repeated virtual screening runs). If the speed of conformer ensemble generation is essential and possible losses in quality are tolerable, less accurate but computationally more efficient approaches may be given preference.

Recently, several studies have been published^11,26,27^ which assessed the performance of well-known commercial and open-source conformer ensemble generators regarding the objectives—accuracy in the reproduction of bioactive conformations, ensemble size, and processing time—using a dataset of 2859 (2912 in ref (26)) high-quality protein-bound ligand conformations extracted from the Protein Data Bank (PDB).^28^ The benchmarking results have shown that commercial and nonfree conformer generators are clearly ahead of open-source tools in all evaluated performance aspects and that substantial improvements on the free software side are required to catch up with leading commercial tools.

Aside from fulfilling the basic quality criteria outlined above, state-of-the-art conformer ensemble generators nowadays have to face an additional challenge imposed by the growing attention flexible macrocyclic systems receive as a new class of promising drug molecules.^29−31^ Macrocyclic molecules do not obey Lipinski’s rule of five^32^ but exhibit interesting and useful properties which set them apart from the mass of typical drug-like small molecules. These are, e.g., improved metabolic stability,^33^ the ability to disrupt protein–protein interactions,^34^ or a higher cellular permeability due to conformational restriction.^35,36^ Furthermore, they are able to bind to proteins which are considered as nondruggable targets due to their lack of hydrophobic cavities which can serve as anchor points for functional groups.^37,38^ Drugs based on macrocyclic scaffolds found widespread clinical application as antibiotics (e.g., rifampicin, vancomycin, macrolides), in cancer therapy,^39−42^ and in immunology and dermatology,^43^ to name a few. When it comes to the generation of representative conformer ensembles of macrocyclic structures, algorithms face the challenge of uniformly searching a huge conformational space in an acceptable amount of time whose size increases dramatically with the number of (partially) rotatable bonds in the macrocycle.^44^ Recently, several studies were published that benchmarked well-known conformer generators regarding their ability to sample macrocycle conformations with enough accuracy and diversity for common CADD applications.^45−47^ The methods employed by these programs can be divided into systematic and stochastic approaches. For example, the conformer generators Omega macrocycle,^48^ MOE LowModeMD,^13^ MacroModel,^49^ Balloon,^21^ and RDKit ETKDG^50^ all pursue a stochastic search strategy. Implementations of systematic sampling approaches are less common, with Conformator,^11^ Prime,^51^ and ForceGen^15,16^ being notable examples in this category. A major algorithmic challenge these programs have to face is imposed by the constrained flexibility of rings, which prohibits an independent rotation of individual ring bonds. Commonly, this problem is handled by cutting one or more bonds of the macrocycle in order to obtain an open-ring equivalent^11^ or multiple nonconnected acyclic parts of the ring,^51^ which can then be sampled in a straightforward systematic way. Afterward, the generated open-ring conformers are evaluated for whether their geometries are suitable for carrying out ring closures. If so, previously cut bonds are reintroduced, and a short 3D structure refinement step is carried out to obtain structurally sound conformers of the original ring system.

In this article, we introduce the new conformer ensemble generation tool CONFORGE (Conformer Generator). CONFORGE is fully open-source (GNU LGPL) and available as part of the Chemical Data Processing Toolkit (CDPKit)^52^ in the form of a versatile command-line tool as well as a set of classes and functions provided by CDPKit’s C++/Python API. CONFORGE aims at delivering state-of-the-art performance for all types of organic molecules in the drug-like chemical space and builds upon proven concepts and algorithms for conformer ensemble generation which were all implemented from scratch with maximum correctness, robustness, and performance in mind. For the computationally efficient and accurate sampling of small-molecule conformers, CONFORGE employs a knowledge-based systematic approach which makes extensive use of pregenerated fragment and torsion angle libraries that were derived from experimental 3D structures. For sampling macrocycle conformers, CONFORGE implements a purely stochastic approach based on DG and force-field-driven structure refinement. The best-suited conformer sampling approach for a processed molecule is either chosen automatically based on the detected presence of a macrocyclic ring system (default behavior) or can be specified by the user in advance for all molecules to process. Furthermore, CONFORGE is able to correctly handle multicomponent molecules like salts and mixtures by the automatic separate generation and later combination of individual component conformer ensembles. CONFORGE’s ability to reproduce experimental 3D structures as well as its computational efficiency and robustness has been assessed thoroughly both for typical drug-like molecules (Platinum Diverse Dataset^26^) and macrocycles (208 macrocyclic structures compiled by Sindhikara et al.^51^). The calculation of various performance metrics and the visual presentation of the obtained results largely follow the established protocol developed by Friedrich et al.^26^ with some meaningful extensions for presenting the macrocycle sampling benchmarking outcome. For reference, several well-known commercial (iCon,^7^ two modes), non-open-source (Conformator,^11^ two modes), and open-source conformer generators (Balloon,^21^ two modes; RDKit,^50^ two variants) were assessed in addition to CONFORGE employing the same benchmarking protocol. The small-molecule benchmarking results have shown (see Results and Discussion) that CONFORGE outperforms all other tested open-source conformer ensemble generators and performs comparably well or better than the commercial generators in terms of both speed of processing and accuracy in the reproduction of bioactive conformations. When it comes to the conformer sampling of macrocyclic structures, CONFORGE is able to reproduce experimental 3D structures with a higher mean accuracy than all other tested generators. To our knowledge, CONFORGE is the first open-source conformer ensemble generator that is able to truly keep up with commercial software with regard to accuracy, speed of processing, applicability, robustness, and ease of use. Given the relevance of conformer ensemble generation as a mandatory preprocessing step for the application of many modern CADD methods, CONFORGE represents a valuable addition to the open-source CADD toolbox that will be highly welcome by the scientific community in the light of the previously present “performance gap”^27^ between open-source and commercial software in this field.

## Methods

The following sections provide insight into the algorithms and methods employed in the implementation of CONFORGE and discuss the design decisions taken to achieve a high performance in the reproduction of bioactive conformations and speed of processing for a wide variety of drug-like organic molecules. The sequence of individual processing steps that must be performed during conformer generation is presented visually by a set of hierarchically linked flowcharts and will be described in detail throughout the text. In general, conformer ensemble generation is a complex endeavor and can fail at nearly all processing stages (e.g., for molecules containing atom types not supported by the force field) or take exceedingly long (e.g., for molecules with many rotatable bonds). Internally, CONFORGE thus has to perform a significant amount of error and timeout checks, which may cause early termination or require intermediate error correction steps. For the sake of simplicity, error and timeout handling have not been incorporated into the flowcharts, and the shown program flow is based upon successful execution of every processing step. Furthermore, in the flowcharts and text, a distinction is made between *compounds* and *molecules* according to the chemical definition of the two terms: A molecule represents a set of covalently bonded atoms where each atom is reachable from any other atom via one or more bonds. Compounds may consist of just one (the common case) or multiple molecules, as is the case for salts or arbitrary mixtures.

CONFORGE has been implemented in C++ and builds on the cheminformatics infrastructure provided by the CDPKit C++ API. For end users, CONFORGE is provided in the form of a command-line tool called “confgen” (Table S6 provides an overview of the supported options) that can be found in the application directory of CDPKit. For developers, CONFORGE’s functionality is also accessible as a set of classes and functions that are part of CDPKit’s C++/Python-API and allow for a seamless integration of CONFORGE into its own CDPKit-based applications.

### Top-Level Conformer Generation Workflow

**Figure 1:**
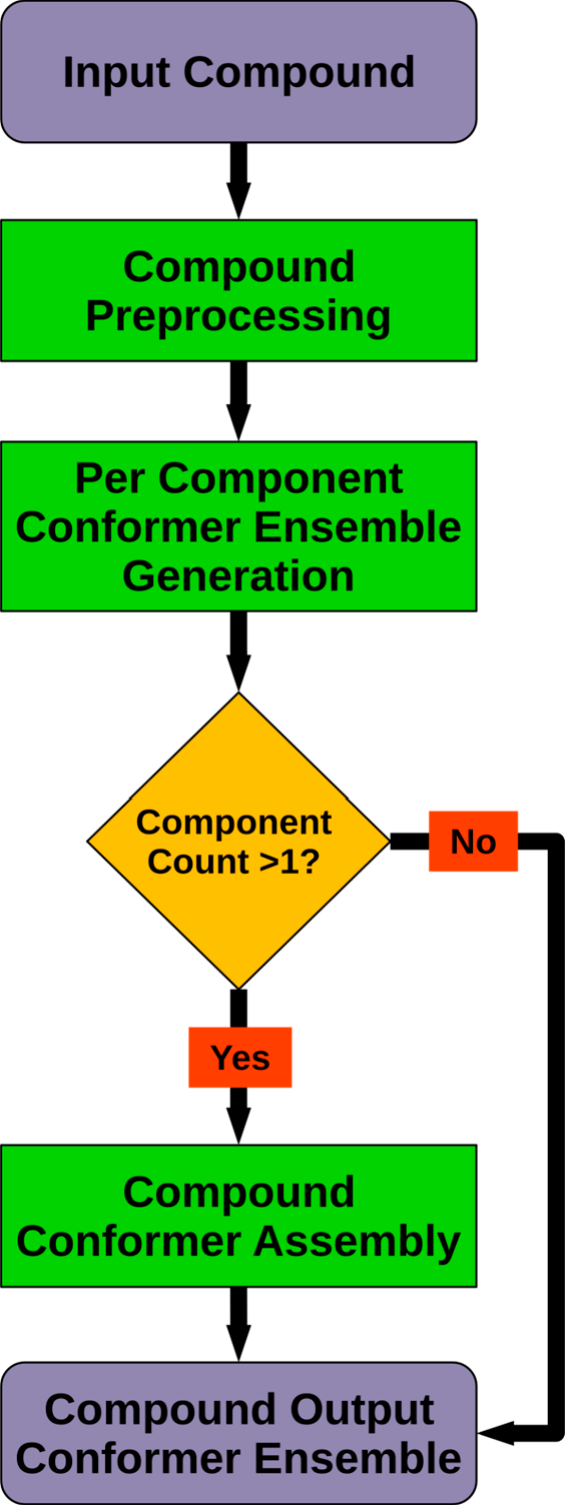
Top-level conformer ensemble generation workflow for a given input compound. Further details on individual processing steps can be found in Figure 2 (Compound Preprocessing), Figure 3 (Per Component Conformer Ensemble Generation), and Figure S4 (Compound Conformer Assembly).

Figure 1 shows the top-level conformer generation workflow executed for an uninitialized input compound (e.g., read from an input file) to obtain a structurally diverse, low-energy output conformer ensemble (conformer energies are estimated by means of a molecular force field). In the first step, preprocessing of the input compound takes place, where the addition of missing hydrogens is performed and required data for the subsequent processing steps are calculated (see Compound Preprocessing for details). For compounds comprising only a single molecule, the conformer ensemble generated in the next step already represents the output conformers of the compound. For multimolecule compounds, CONFORGE generates a separate conformer ensemble for every molecule and then arranges combinations of selected molecule conformers in 3D space to obtain the final compound output conformers (see Multi-Molecule Output Conformer Ensemble Generation in the Supporting Information (SI)).

### Compound Preprocessing

**Figure 2:**
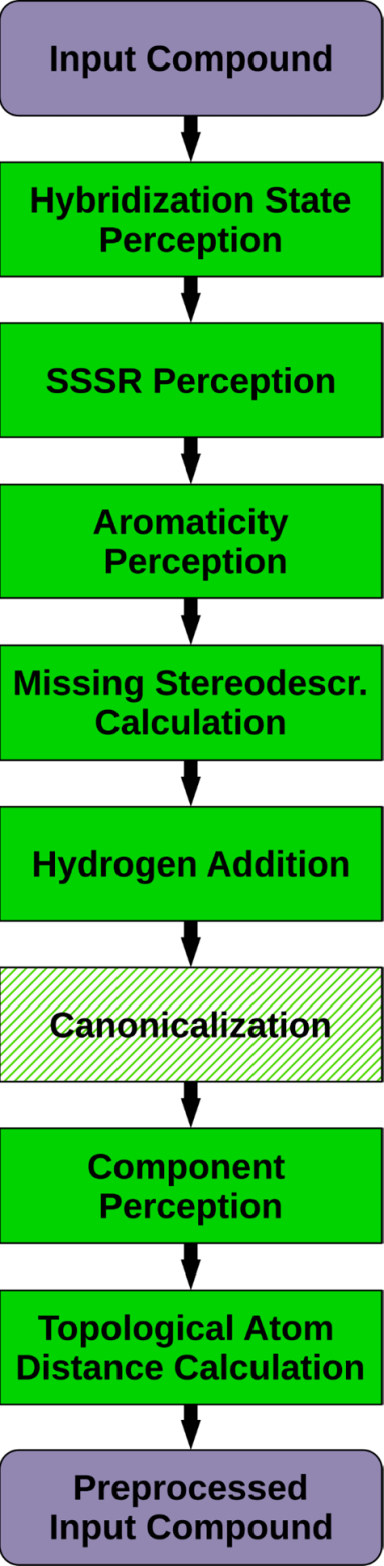
Sequence of processing steps carried out to prepare an input compound for conformer ensemble generation.

Compound preprocessing (Figure 2) comprises several substeps that prepare the raw input compound and calculate various molecular properties required for the generation of proper 3D structures and conformer ensembles. In the first step, the hybridization state of every atom is determined from the atom type, formal charge, and number and order of incident bonds. In the next step, the smallest set of smallest rings (SSSR) is perceived, which, together with the previously determined atomic hybridization states, allows identification of atoms and bonds that are part of planar aromatic ring systems.

In general, stereo configurations of chiral atoms and asymmetric double bonds that were specified in the input data (e.g., a SMILES string) are retained and will be considered accordingly in the 3D structure generation process. For atoms and bonds with undefined stereochemistry, an attempt is made to calculate missing stereo descriptors from supplied 3D atom coordinates. If 3D atom coordinates are not available, the configuration of chiral atoms is left unspecified, and the selection of suitable configurations is delegated to later processing steps. For asymmetric double bonds with no predefined stereochemistry, a *trans* configuration of the sterically most bulky substituents on either side of the double bond is selected. To efficiently estimate the steric bulk of substituents, we use a modified version of the Morgan algorithm^53^ that stops after the iterative calculation of atom connectivity values (CVs). The higher the CV of an atom, the more complex its neighborhood is, and the more space-consuming the substituent it represents will presumably be.

The next processing step adds hydrogens onto heavy atoms considered to be missing according to the associated chemical element’s characteristic valence state(s) and specified formal charge. It is worth noting that this preprocessing step does not affect the protonation state (present at a particular pH value) of the heavy atoms in any way. Here only a “conversion” of implicitly present hydrogens to an explicit representation is carried out.

CONFORGE employs a DG-based approach^24,25^ whenever 3D structures need to be generated from scratch. Here, randomly distributed starting atom positions are iteratively refined until a valid local energetic minimum structure has been obtained. Which of the energetic minimum conformers is eventually obtained heavily depends on the order of atoms during the initial assignment of random atom positions. To guarantee input atom order-invariant generation of output conformer ensembles, the connection tables of input compounds need to be canonicalized before any conformers are generated. This canonicalization step is optional and has to be explicitly enabled by the user if slight output differences for structurally identical input compounds with varying atom orders are not acceptable. For the connection table canonicalization task, CONFORGE employs an implementation of the algorithm devised by McKay.^54^

After (optional) compound canonicalization, a perception of structurally separated compound components (molecules) is carried out. Components are identified by performing a depth-first search for all atoms reachable via a bond path from a given start atom. Found reachable atoms and the start atom belong to the same component and are marked accordingly. The search procedure is then repeated for the next not yet visited atom in the atom list until no more unvisited atoms are left.

In the last compound preprocessing step, a topological distance matrix is generated for every component of the input compound. Topological atom distances are needed to generate DG constraints and for the parametrization of Merck Molecular Force Field 94 (MMFF94) van der Waals interactions.^55^ To determine topological distances, we employ a breadth-first search approach, where the length of the bond path from a given start atom to a reachable atom is noted in the corresponding cells of the distance matrix. Alternatively, we also implemented the Floyd–Warshall algorithm^56^ to determine the topological distances. However, the Floyd–Warshall implementation was significantly slower than the breadth-first search approach and eventually gave up in favor of the latter.

### Molecule Conformer Ensemble Generation

**Figure 3:**
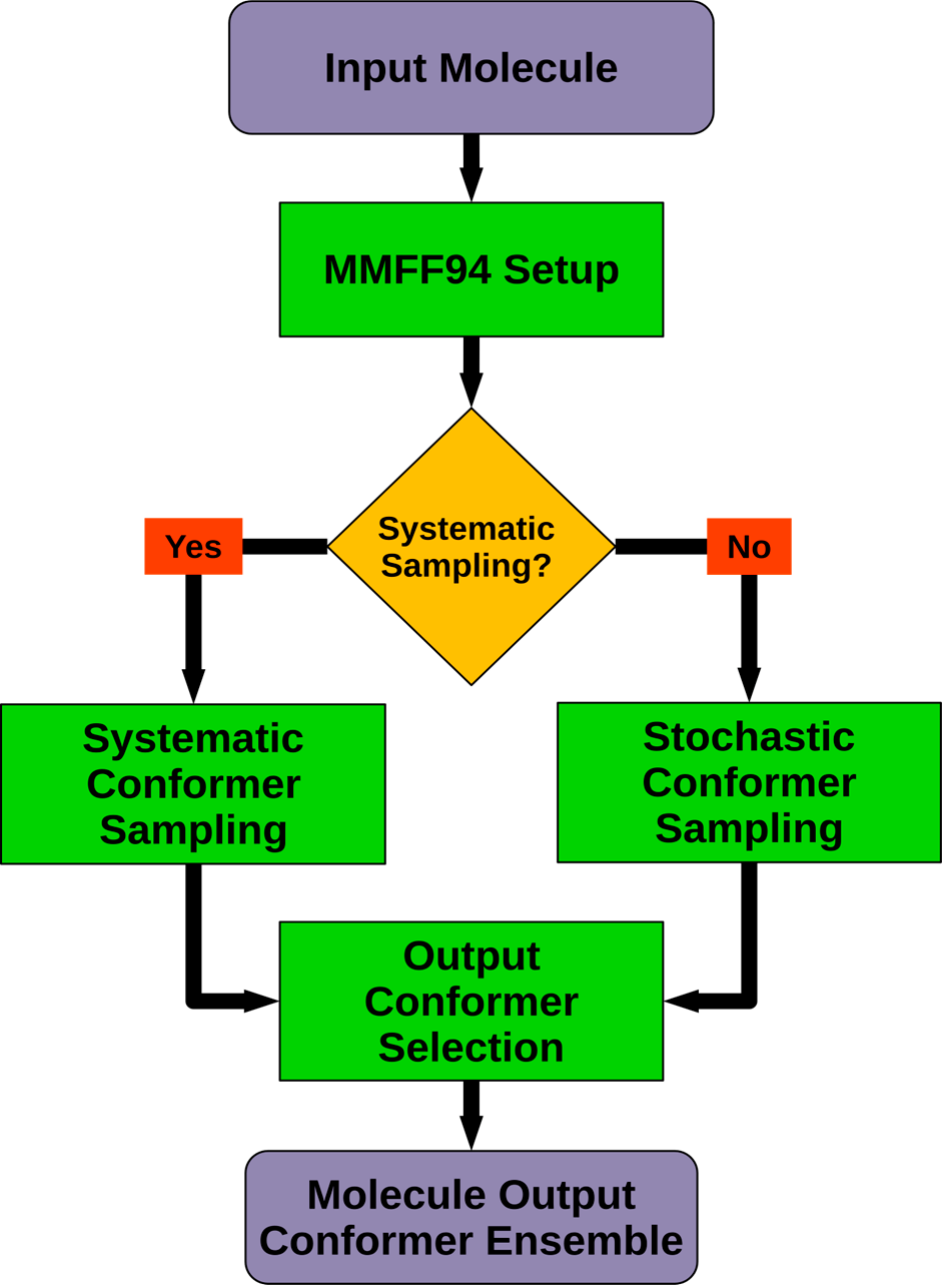
Molecule conformer ensemble generation workflow. Further details on individual processing steps can be found in Figure 4 (Stochastic Conformer Sampling), Figure 6 (Systematic Conformer Sampling), and Figure 9 (Output Conformer Selection).

As already noted, a separate molecule conformer ensemble is generated for every component of the input compound. The molecule conformer generation workflow shown in Figure 3 may thus be executed more than once depending on the number of components that could be identified in the compound preprocessing stage.

The conformer generation process for a given input molecule starts with the perception and parametrization of MMFF94 interactions. Determined force field interactions and parameters will be required for 3D structure refinement and conformer energy estimation in later processing stages. CONFORGE utilizes CDPKit’s full-featured MMFF94 implementation, whose correctness has been thoroughly validated in all aspects using the datasets provided by the MMFF94 Validation Suite.^57^ In the next step, the ultimate decision is made whether to use a systematic or stochastic sampling approach for generating the molecule conformers. The systematic approach (see Systematic Conformer Sampling) performs best for typical drug-like molecules composed of chains and relatively rigid ring systems. Stochastic sampling (see the next section) has proven to be advantageous for structures comprising flexible macrocyclic rings, where conformers cannot be sampled by means of simple systematic torsion driving due to the present rotational restrictions. By default, CONFORGE selects a stochastic sampling approach whenever the SSSR of the molecule contains a ring that incorporates more than 10 nonaromatic single bonds. The automatic selection of a suitable sampling method can be overridden by specifying the method to use in advance in the CONFORGE settings. Once a pool of output conformer candidates has been generated by the chosen method, a final output conformer ensemble is compiled in the last processing step of the workflow. Which, and how many, of the candidate conformers end up in the output conformer ensemble depend on various user-specified settings which control allowed energy range, desired structural diversity, and maximum ensemble size. Details regarding the output conformer selection process can be found in Molecule Output Conformer Ensemble Compilation.

### Stochastic Conformer Sampling

**Figure 4:**
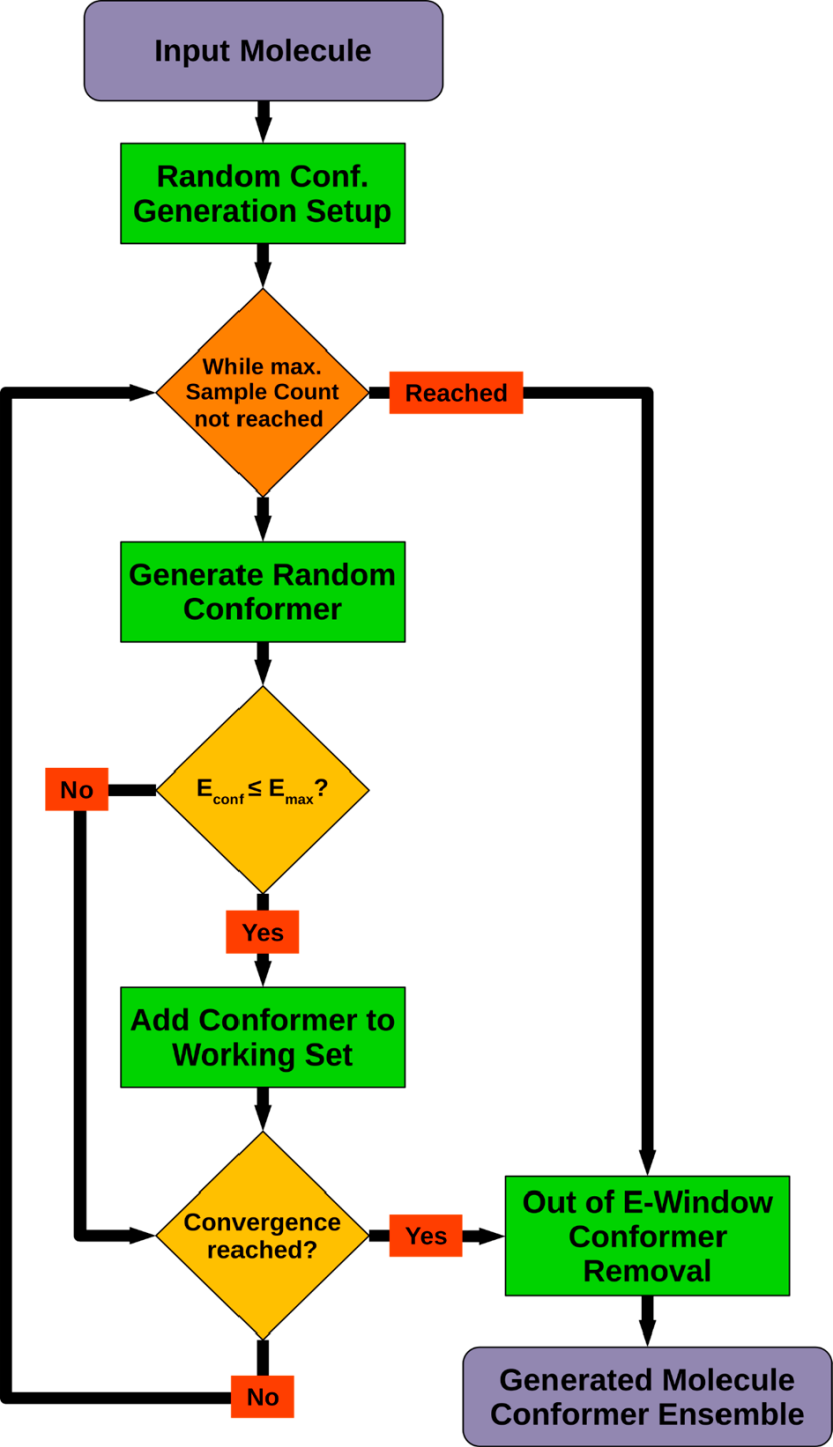
Stochastic conformer sampling workflow. *E*_conf_ = conformer energy; *E*_max_ = conformer energy threshold according to the current lowest-energy conformer and energy window setting. Further details on the random conformer generation step can be found in Figure 5.

Stochastic conformer sampling is a relatively simple but time-consuming process, which is based on the assumption that randomly generated structurally diverse low-energy conformers will be uniformly distributed over the whole conformational space of a molecule and that a sufficiently large set of such samples will represent a large fraction of the energetically most favorable torsion angle combinations. Figure 4 shows the principal steps of the stochastic conformer sampling workflow, as implemented in CONFORGE. The first step is concerned with initializing the random conformer generation unit. Random low-energy conformers are generated by a DG-based approach, where, in accordance with predefined atom distance and volume constraints, initially randomly distributed atom positions are successively refined until a valid energy-minimized 3D structure of the molecule is obtained (see the next section for details). During the sampling process, random conformers are generated iteratively until a specified maximum number of conformers have been sampled (default: 2000 conformers), the granted sampling time has been exceeded, or the generation of new unique conformers has ceased (convergence reached). Within the sampling loop, each newly generated conformer is first tested whether its energy is lower than or equal to the current energy threshold. The energy threshold equals the lowest conformer energy encountered thus far plus the specified energy window size. If the generated conformer passes the energy check, it is added to the working ensemble; otherwise, it gets discarded. Next, a sampling convergence check is carried out which decides whether the conformational space has been sampled densely enough and therefore the sampling loop may already be exited. In the convergence check, after a certain number of conformer generation cycles (default: 100 cycles) have passed, the number of newly generated unique conformers is determined. This is done by first performing an energy-based removal of duplicate conformers (conformers with an energy difference of ≤0.01 kcal/mol) from the working ensemble and then comparing the number of remaining conformers with the ensemble size obtained after the last duplicate removal step. If the ensemble size did not increase since the last check, the sampling process has converged and can be stopped. After leaving the iterative sampling loop, any conformers in the working ensemble with energies outside the energy window (see above) get discarded, and the stochastic sampling process terminates.

### Random Conformer Generation

**Figure 5:**
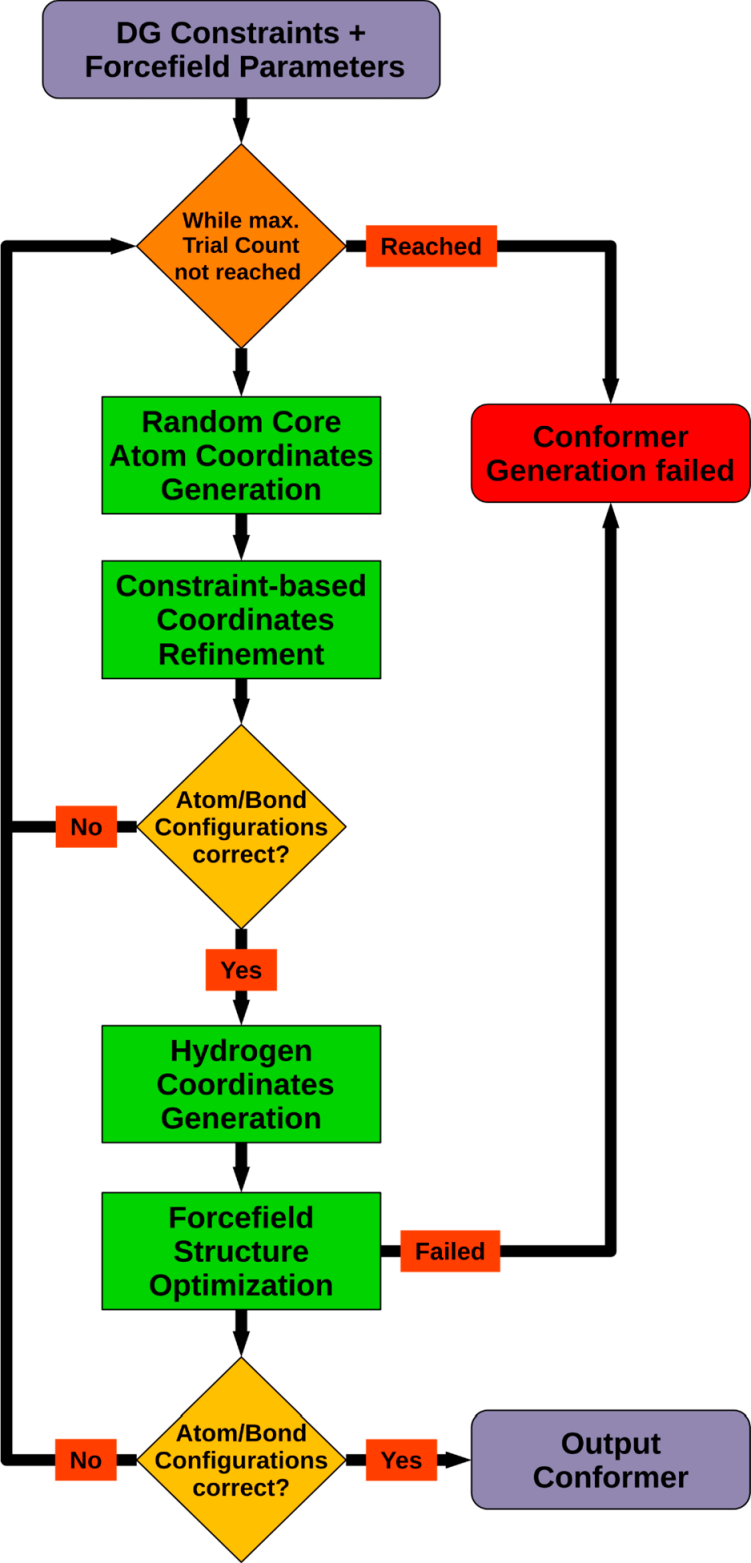
Random conformer generation workflow based on DG and subsequent force-field-based structure optimization.

CONFORGE uses the random conformer generation functionality whenever arbitrary low-energy 3D structures of a molecule have to be generated from basic molecular graph information only (e.g., for stochastic conformer sampling, see previous section). The generation of a single random 3D structure comprises the following sequence of steps (Figure 5).

First, the 3D coordinates of all heavy atoms and any hydrogens which are connected to stereocenters with defined configuration are initialized with random values lying in a range equaling the atom count times a structure-type-specific factor (e.g., 0.5 for macrocycle sampling). Hydrogens usually represent the majority of the atoms in typical organic molecules, and their exclusion allows for a significant speedup of the subsequent DG-based raw 3D structure generation step. Here, the initial random atom positions are optimized by a coordinate embedding procedure^58^ until they meet certain pairwise distance and tetrahedral volume constraints. The geometric distance range constraint assigned to an atom pair *p*_*ij*_ = (*a*_*i*_, *a*_*j*_) depends on its topological distance TD_*ij*_: For directly bonded atoms (TD_*ij*_ = 1) the upper and lower distance limits are set to the MMFF94 bond length taken from the assigned bond stretching interaction parameters. The upper and lower distance limits for a geminal atom pair (TD_*ij*_ = 2) are calculated from the respective MMFF bond lengths and the equilibrium bond angle specified by the assigned bond stretching and angle bending interaction parameters. For vicinal atom pairs (TD_*ij*_ = 3), where the central bond is not a double bond with a defined configuration, the lower limit is set to the calculated distance of the atoms in a coplanar arrangement and the upper limit to the distance in an anti-coplanar arrangement, taking into account the MMFF94 bond lengths and angles of the involved bonds. Vicinal atoms connected to a central double bond with a defined configuration are assigned a fixed distance corresponding to the spacing of the atoms in a coplanar arrangement if the central bond’s configuration is *cis* (with regard to the atom pair) and the corresponding distance in an anti-coplanar arrangement if the configuration is *trans*. All remaining atom pairs (TD_*ij*_ > 3) are assigned a lower distance limit, calculated as the sum of the covalent atom radii plus an additional safety spacing of 1.5 Å, and an upper limit equal to the total sum of all MMFF94 bond lengths. Volume constraints are generated for atoms bonded to tetrahedral stereogenic centers and for groups with known planar atom arrangements. For stereogenic centers, the sign of the volume spanned by the neighboring atoms depends on the specified configuration and is enforced by setting the corresponding volume range limits to ±0.5 and ±1000 Å^3^ (empirical values), respectively. Planar groups are, for example, formed by amide nitrogens and sp^2^-hybridized or aromatic atoms with three incident bonds and furthermore by atoms connected to amide, aromatic, and double bonds. To enforce a planar arrangement of such atom groups, the upper and lower volume range limits are set to zero.

Once a raw 3D structure has been obtained from the embedding process, a first check is made whether the configurations of defined stereogenic centers are correct with respect to the generated coordinates. If the check fails for at least one stereocenter, then a new attempt is made to generate a valid raw structure starting from a different set of random atom positions. If after a certain number of trials (default: 10) still no valid structure could be obtained, the random conformer generation procedure terminates and reports an error. Otherwise, processing continues with the next step, where the coordinates of removed hydrogens are calculated according to the hybridization state of the connected heavy atoms and already assigned atom positions. After that, the geometry of the hydrogen-complete raw 3D structure is further refined by iterative minimization of its MMFF94 energy until the energy gradient norm or the change of energy is below a certain target threshold (the stopping criterion and threshold value have to be specified by the caller and are context-dependent). For energy minimization, the algorithm devised by Broyden, Fletcher, Goldfarb, and Shanno (BFGS)^59−62^ is used. The CDPKit implementation of the BFGS algorithm is based on code taken from the GNU Scientific Library (GSL).^63^ If the energy-driven structure refinement procedure fails, then the random conformer generation procedure terminates immediately and reports an error. In the final step of the workflow, all defined stereocenters are once again checked for the correctness of their configurations. If no errors are found, the refined set of atom coordinates is output, and the workflow terminates with success. Otherwise, as described previously, a new attempt to obtain a valid 3D structure will be made.

### Systematic Conformer Sampling

CONFORGE performs systematic conformer sampling by applying different combinations of torsion angles at the rotatable bonds of the processed molecule on beforehand-generated conformer 3D structure templates. Compared with stochastic sampling, the systematic approach usually demands much less processing time for small drug-like molecules since all conformers of a given 3D structure template can be generated by relatively fast rigid-body transformations instead of having to repeatedly perform a time-consuming *ab initio* generation of energy-minimized random conformers.

For the construction of 3D structure templates, CONFORGE employs a fragment-based approach in which the overall 3D structure is assembled from smaller structural building blocks such as chain fragments and ring systems. The idea behind this approach is to pregenerate reasonable 3D structures of frequently occurring molecular fragments by an external program (“genfraglib”, which can be found in the application directory of CDPKit) and store the resulting atom 3D coordinate sets in a permanent library for later use. Via this strategy, a considerable speedup of the 3D structure template buildup process can be achieved since time-consuming *ab initio* generation of 3D coordinates (see Random Conformer Generation) can be circumvented for most fragments found in typical drug-like organic molecules.

**Figure 6:**
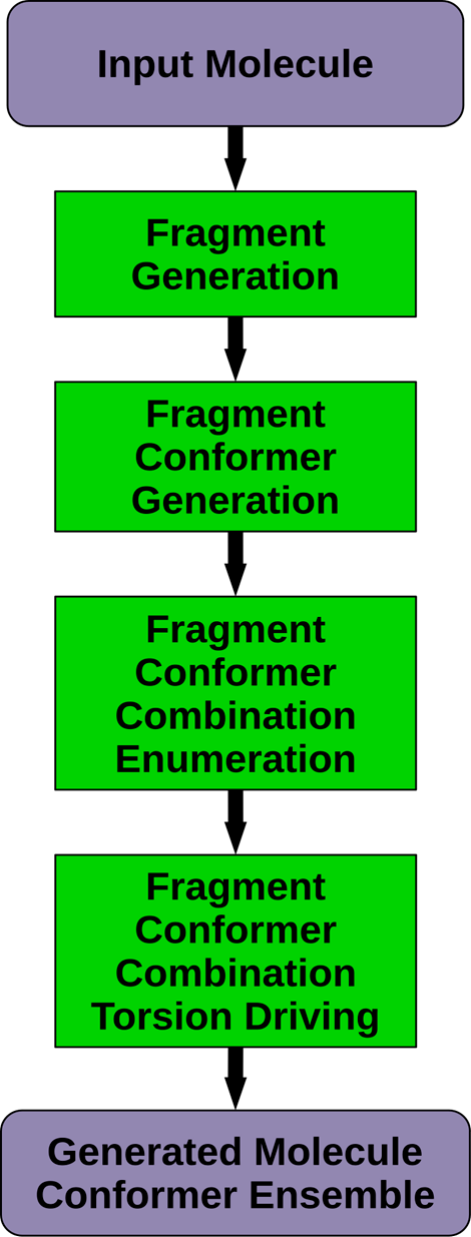
Systematic conformer sampling workflow. Further details on individual processing steps can be found in Figure S1 (Fragment Generation), Figure S2 (Fragment Conformer Generation), and Figure 7 (Fragment Conformer Combination Torsion Driving).

Figure 6 illustrates the major processing steps performed for a given input molecule in the implemented systematic conformer sampling workflow. In the first step, the provided molecule is broken down into smaller structural building blocks by cutting specific bonds that have been identified by a set of fragmentation rules (see Molecule Fragmentation in the SI). Afterward a conformer ensemble is generated for each obtained fragment, which, depending on conformational flexibility, may consist only of a single 3D structure or multiple conformers. For rigid fragments such as purely aromatic ring systems and chains/moieties lacking rotatable bonds, a single 3D structure is generated. Chains comprising rotatable bonds and flexible ring systems are sampled more thoroughly until a representative set of low-energy conformers has been obtained. As pointed out before, for each processed fragment, an attempt is made to obtain pregenerated 3D coordinates from an on-disk fragment library to speed up the overall conformer generation process. If a suitable library entry is not available, conformers will be generated on the fly using CONFORGE’s random conformer generation (see the previous section) and torsion driving functionality. More details on fragment library lookup, on-the-fly conformer generation, and fragment conformer postprocessing can be found in the SI. In the next step, a set of fragment conformer combinations (FCCs) is generated. FCCs are represented by *N*-tuples of fragment conformer indices, where *N* corresponds to the total number of resulting input molecule fragments. The energy of an FCC is calculated as the sum of the MMFF94 energies of the fragment conformers referenced by the index-tuple. To prevent the exhaustive consumption of main memory and an exaggerated processing time by very high numbers of possible FCCs (e.g., for larger peptides), the FCC generation process stops once a certain number of stored FCCs has been reached (100,000 in the current implementation). Furthermore, only FCCs with energies lower that or equal to the calculated energy threshold are stored for further processing. The energy threshold corresponds to the minimum FCC energy plus the specified energy window size times the safety factor of 1.5.

In the final step of the workflow, each FCC serves as a 3D structure template for the generation of derived molecule conformers by systematic torsion driving at rotatable bonds linking the fragments. By means of this divide-and-conquer strategy, it is possible to sample a representative share of the lowest-energy conformers in an acceptable amount of time, even in the case of large and flexible molecules. Further details on the assignment of torsion angles to rotatable bonds and the implementation of the torsion driving process can be found in Fragment Conformer Combination Torsion Driving.

### Fragment Conformer Combination Torsion Driving

This processing step represents the heart of the systematic conformer sampling workflow (see Systematic Conformer Sampling) and produces an ensemble of output conformers from a set of energy-ordered input FCCs. 3D structures of the output conformers are generated by a torsion driving procedure that aligns individual fragment conformers specified by the processed input FCCs along rotatable bonds linking the fragments (see Molecule Fragmentation in the SI). The relative spatial orientation of the aligned fragment conformers is dictated by rotatable-bond-specific dihedral angles, which are retrieved from matching torsion library entries. Depending on the number of distinct torsion angle combinations possible for the present set of rotor bonds, a corresponding number of output conformers will usually be generated for each processed FCC. For big and quite flexible molecules, the total number of possible conformers can quickly reach rather high figures, and an attempt to generate all of these conformers will fail due to excessive main memory and processing time consumption. However, carrying out the processing of FCCs in order of increasing energy and the early pruning of high-energy conformers during torsion driving ensures that also in cases where the conformational space cannot be explored exhaustively, most of the low-energy conformers will be captured and end up in the generated output ensembles (see the Results and Discussion).

**Figure 7:**
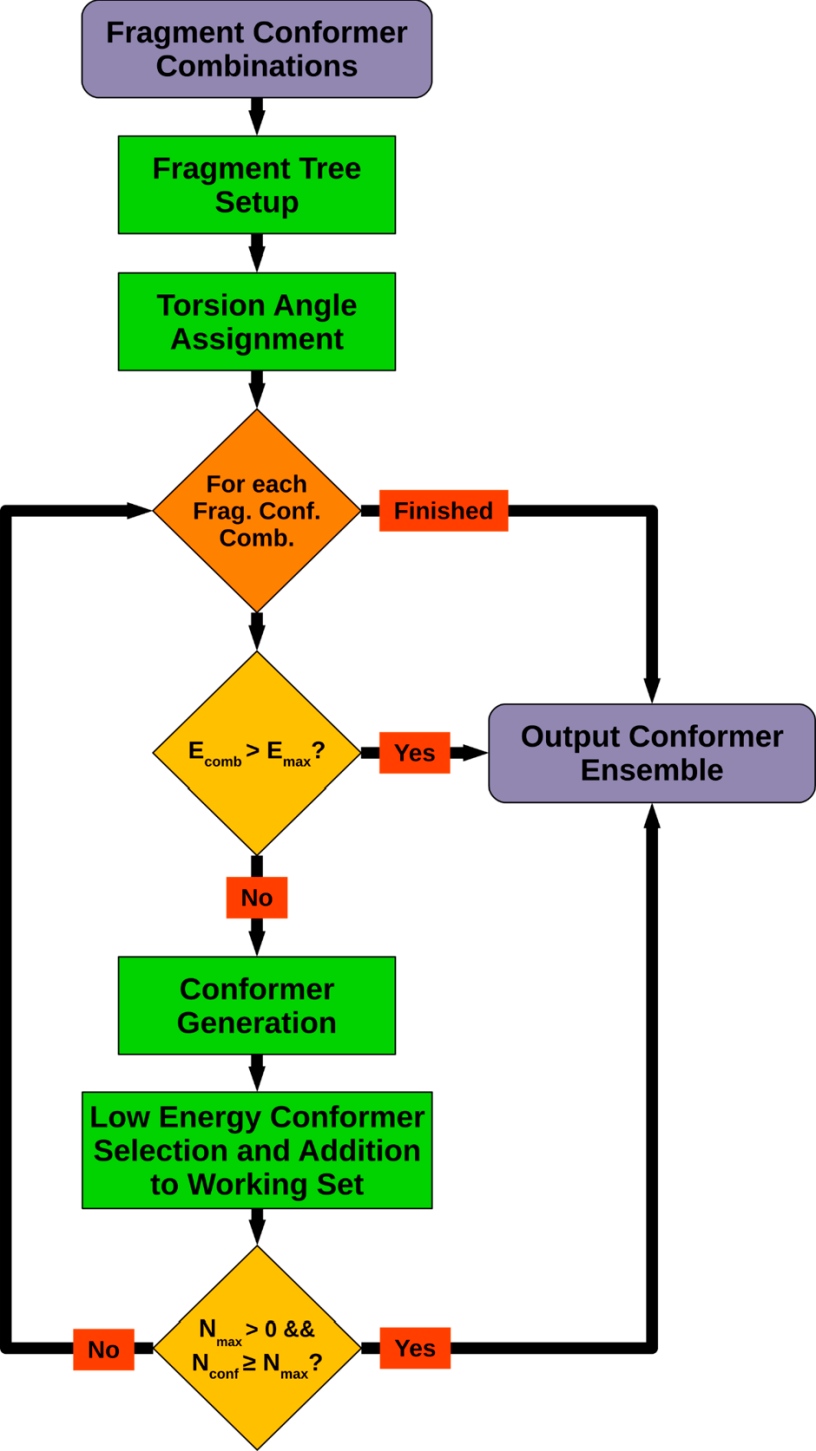
Fragment conformer combination torsion driving workflow. *E*_comb_ = sum of fragment conformer energies of the current combination; *E*_max_ = upper energy limit according to the energy of the combination that led to the current lowest-energy conformer and the energy window setting; *N*_conf_ = current number of generated output conformers; *N*_max_ = maximum number of output conformers.

**Figure 8:**
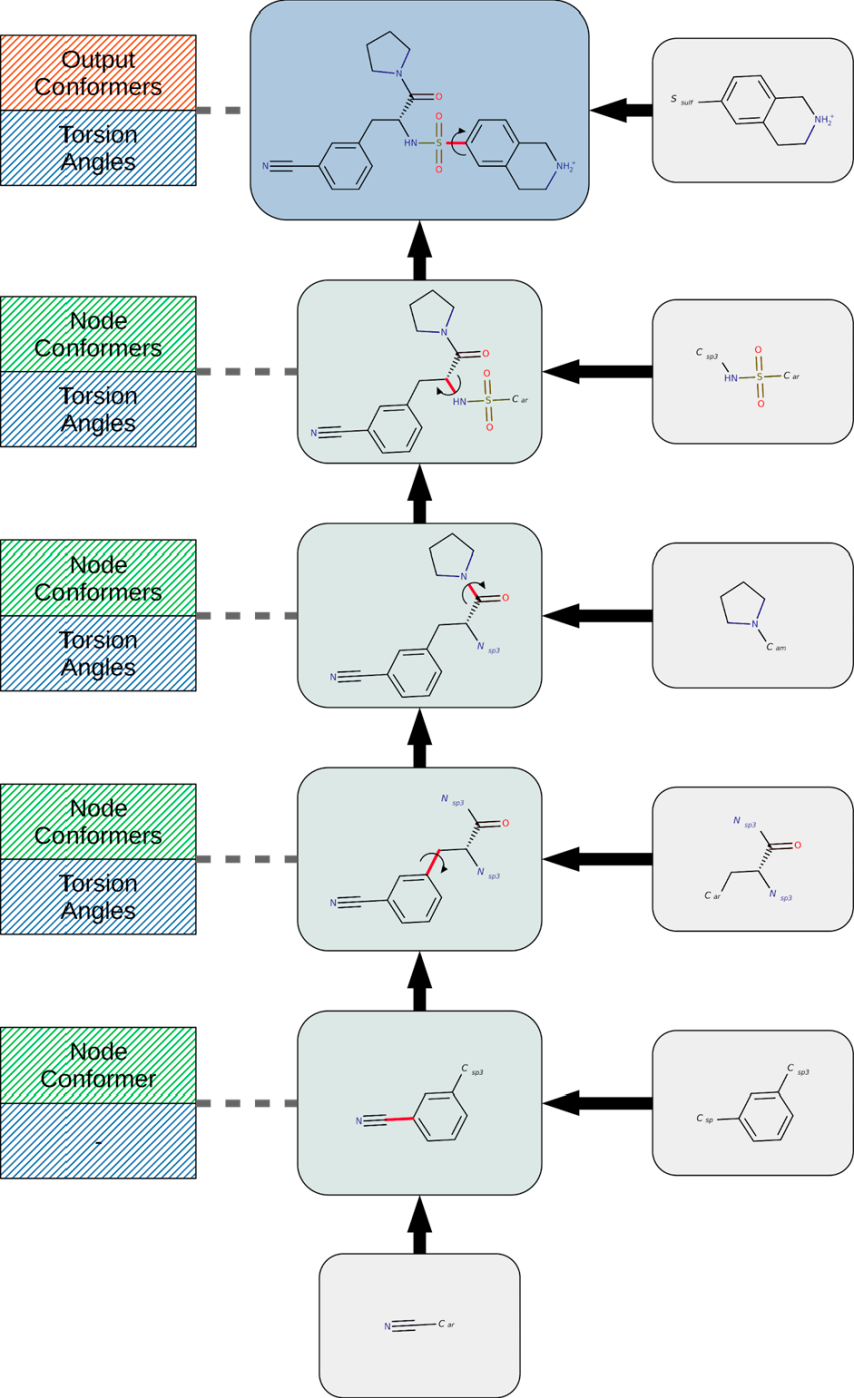
Example of a fragment tree built for the molecule shown in the root node (top of the figure). This data structure serves as the basis for the systematic generation of molecule conformers by performing torsion driving on a given FCC (represented by the fragment conformers stored in the leaf nodes). For every rotatable bond of the molecule (marked in red), a dedicated internal node stores the conformers that can be generated by twisting all possible child node conformer combinations around the rotatable bond according to the dihedral angles provided by the assigned torsion library entry.

The first step of the overall torsion driving workflow (Figure 7) is concerned with the setup of the fragment tree data structure. This data structure represents the in-memory foundation of the torsion driving algorithm and comprises a set of linked nodes organized in a treelike manner. Each nonleaf node of the tree references a particular rotatable bond and provides storage for conformers that can be generated by aligning all possible pairs of child node conformers along the referenced bond applying the dihedral angles specified by an assigned torsion library entry. The leaf nodes are associated with the fragments constituting the parent structure (root node), and each stores a particular fragment conformer as specified by the currently processed FCC. For the sake of better understanding, Figure 8 shows an example of a typical fragment tree constructed by the setup procedure for the molecule shown in the root node.

Once the fragment tree data structure has been set up, an assignment of proper dihedral angles for the rotatable bonds referenced by the nonleaf nodes is carried out. Here, for each rotatable bond, a lookup for a matching entry in the loaded torsion libraries is performed. Torsion library entries specify matching rotatable bonds by a single SMARTS pattern^64^ which describes a linear path of three or four atoms and provides lists of preferred dihedral angles and associated tolerance intervals. The torsion library used by CONFORGE has been derived from the collection of rotatable bond patterns and corresponding dihedral angles compiled by Schärfer et al.^65^ and Guba et al.,^66^ which resulted from statistical analyses of torsion angle preferences in the drug-like chemical space. Based on the set of rotatable bond patterns in the most recent version of the library by Guba et al., we performed our own torsion angle analysis using a database of experimentally determined receptor-bound ligand 3D structures extracted from the PDB.^67^ Based on the obtained results, several new library entries have been added, and various modifications to the angle and tolerance lists of existing entries in the library by Guba et al. have been made. Aside from the built-in library, CONFORGE also supports multiple user-specified external torsion libraries, which are included in the search for matching entries. These will take precedence over the entries in the built-in library and thus enable customization of the dihedral angles used in the torsion driving core procedure.

Depending on the conformer generation settings in effect and the presence of local symmetry, dihedral angles provided by the looked-up library entries may undergo further processing before they are assigned to the corresponding tree nodes: (i) In the case of rotatable bonds which represent an axis of one-, two- or threefold rotational symmetry of at least one of the connected fragments, all redundant angles that would lead to the generation of conformer duplicates are removed. Often-occurring fragments with known rotational symmetry (e.g., trifluormethyl, phenyl, *tert*-butyl, etc.) have been tabulated as a list of SMARTS patterns and are identified at runtime by substructure searching. (ii) If torsion angle tolerance range sampling has been enabled (default: not enabled), two additional angles per listed dihedral angle will be calculated that mark the beginning and end of the dihedral angle’s first tolerance range (see ref (66) for the definition of the first tolerance range).

After the torsion angle assignment step, the workflow enters a loop in which a set of output conformer candidates are generated for each specified input FCC. The loop is exited either after all FCCs have been processed or if one of the two early exit conditions is fulfilled. The first early exit check is carried out before entering the torsion driving core procedure and evaluates whether the energy of the currently processed FCC is above a certain upper energy limit. The energy limit is calculated as the sum of the energy of the FCC that resulted in the lowest-energy output conformer generated so far and the specified energy window size. A generation of novel low-energy output conformers from FCCs above this energy limit is very unlikely, and the processing of additional input FCCs will have little impact on the final output conformer ensemble. Hence, the main loop can already be exited at this point with, on average, only small losses in output conformer ensemble quality.

If the FCC passes the energy limit check, then the torsion driving core procedure is entered. Herein, the leaf nodes of the fragment tree are first initialized with the fragment conformers specified by the newly processed FCC, and then a recursive generation of conformers at the nonleaf nodes is carried out. The conformers of a nonleaf node are generated by overlaying pairs of left and right child node conformers at the referenced rotatable bond and then applying rotations to the coordinates of one child conformer according to the torsion angles assigned during setup. This process is repeated for all possible child node conformer pair and torsion angle combinations and finally results in a set of conformers that represents the conformational space of the molecule substructure covered by the fragments referenced in the subtree rooting at the currently processed node.

The MMFF94 energy *E*_A–B_ of a conformer A–B that was generated from two child node conformers A and B for the torsion angle Θ can be calculated according to eq 1:
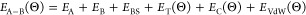
1where *E*_A_ and *E*_B_ are the MMFF94 energies of the child node conformers A and B, respectively, *E*_BS_ is the bond stretching interaction energy of the rotatable bond, *E*_T_ is the sum of the energies of torsion interactions involving the rotatable bond, *E*_C_ is the sum of the energies of electrostatic interactions between the child node substructures, and *E*_VdW_ is the sum of the corresponding van der Waals interaction energies. As can be seen from eq 1, *E*_A_, *E*_B_, and *E*_BS_ are torsion-angle-independent constants whose values, once calculated, can be reused in the calculation of other energy terms in which they are involved. The torsion driving procedure exploits this fact and is thus able to perform a fast energy calculation of newly generated conformers since every parametrized force field interaction energy term, in the worst case, needs to be evaluated only once per generated conformer.

For each newly generated conformer, a first check is made to determine whether its energy exceeds the current energy threshold, which equals the sum of the minimum conformer energy encountered so far and the specified energy window size. If so, then the generated conformer is discarded. Otherwise, it is added to the node’s current working set. After all possible conformers of a node have been generated, each saved conformer is again checked to see whether its energy is within the allowed energy window, and if not, it gets discarded. Finally, the remaining conformers are ordered by increasing energy, and if the number of conformers exceeds the maximum allowed pool size (default value: 10,000 conformers), the number of conformers is reduced by pruning excessive high-energy conformers. These postprocessing steps ensure that potential high-energy molecule conformers get identified and removed from the processing pipeline already early on, and in the case of molecules comprising many fragments and/or high numbers of torsion angles per rotor bond, a combinatorial explosion of live conformers is avoided.

After the bottom-up generation of conformers has finished at the root node of the fragment tree, the torsion driving procedure terminates. The root node now stores a set of final low-energy molecule conformers derived from the currently processed FCC. For each conformer, a check is then made whether its energy is above an upper limit calculated as the sum of the minimum conformer energy encountered so far for all processed FCCs and the specified energy window size. If the conformer’s energy is above the threshold, then the conformer gets discarded. Otherwise, it is added to the output conformer working set. If during the processing of the root node conformer ensemble a new energetic minimum conformer is encountered, the energetic minimum gets adapted, and all conformers in the current working set that are now above the new upper energy limit are discarded.

After all conformers have been processed, a check is made whether the second early loop exit condition is met. The condition will be fulfilled if the number of conformers in the working set is greater than or equal to a specified nonzero maximum pool size (default value: 10,000 conformers). If so, the loop will be exited, and the torsion driving workflow terminates. Otherwise, the next input FCC (if available) is processed as described above.

### Molecule Output Conformer Ensemble Compilation

**Figure 9:**
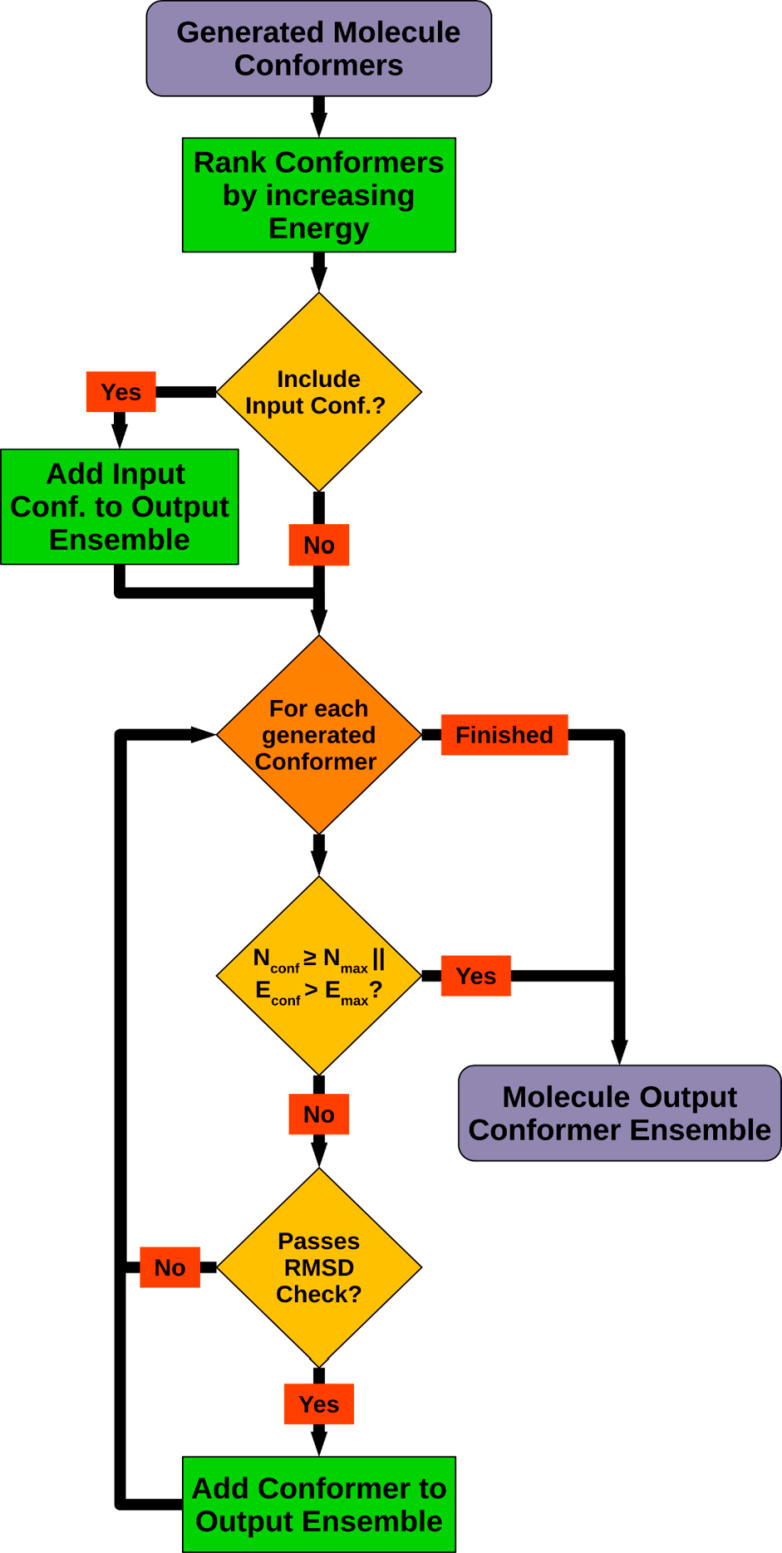
Molecule output conformer ensemble compilation workflow based on conformer energy ranking and pairwise RMSD calculations. *E*_conf_ = conformer energy; *E*_max_ = upper conformer energy limit according to the current lowest-energy conformer and energy window setting; *N*_conf_ = current number of generated output conformers; *N*_max_ = maximum number of output conformers.

Sets of low-energy molecule conformers obtained from the systematic or stochastic sampling procedures usually do not yet fulfill all user-specified output ensemble characteristics (structural diversity, maximum output ensemble size and energy window size) and may contain a significant number of duplicates or clusters of structurally too similar conformers. Figure 9 shows the workflow of the postprocessing procedure which takes care of compiling a final output conformer ensemble with desired characteristics from a set of supplied input conformers. The procedure starts with ranking the supplied input conformers by increasing the energy to ensure a preference for low-energy conformers in the later output conformer picking stage. Afterward, a check is made whether a present 3D structure of the molecule that has been provided with the processed molecule on input shall be added to the output conformer ensemble (default setting: not included). If so, the input 3D structure gets extracted, and after its MMFF94 energy is calculated, it is added to the output conformer working set.

Processing then enters the main section of the workflow, where the final output conformers are selected iteratively from the energy-ordered set of input conformers based on mutual structural dissimilarity. The picking loop exits if either all input conformers have been processed, the maximum output ensemble size has been reached, or the energy of the currently processed conformer exceeds the energy limit, which is calculated as the sum of the minimum conformer energy (equal to the energy of the first conformer) and the specified energy window size. Within the loop, a processed input conformer is selected as an output conformer only if the heavy-atom RMSD between the current conformer and any of the previously selected output conformers is not below a specified threshold value (default setting: 0.5 Å; note: in order to retain conformers of terminal heteroatom hydrogen rotor bonds, it is recommended to lower the RMSD threshold accordingly). The RMSD of an evaluated conformer pair is calculated by performing RMSD minimizing 3D alignments using CDPKit’s implementation of the Kabsch algorithm^68,69^ for all possible homomorphic atom mappings that might result from the presence of topological symmetry. To avoid excessive memory and processing time consumption in the case of highly symmetric molecules, the number of processed mappings has been limited to a hardcoded maximum value of 131,072 (note: if this limit is reached, which happens rarely in practice, the output ensemble may contain conformer pairs with an RMSD below the specified threshold).

When the RMSD value resulting from one of the performed 3D alignments falls below the specified threshold, further processing stops, and the evaluated input conformer gets rejected. Otherwise, if all performed pairwise RMSD checks have been passed successfully, the conformer is added to the output conformer ensemble, and processing continues with the next input conformer in line.

Although the implemented output conformer selection algorithm is quite simple, the obtained ensembles nevertheless fulfill all major quality criteria, which is supported by the benchmarking results presented in the Results and Discussion.

## Results and Discussion

The ability of CONFORGE to reproduce experimental 3D structures and its computational efficiency and robustness have been thoroughly assessed for both typical drug-like molecules and macrocyclic structures. The calculation of various performance metrics and the visual presentation of the obtained results largely follows the established protocol developed by Friedrich et al.^26^ with some meaningful additions for the presentation of the macrocycle sampling benchmarks. For reference, several well-known commercial (iCon,^7^ two modes), non-open-source (Conformator,^11^ two modes), and open-source conformer generators (Balloon,^21^ two modes; RDKit,^50^ two variants) were assessed in addition to CONFORGE employing the same benchmarking protocol. The corresponding results will be presented and discussed along with the ones obtained for CONFORGE in the next sections.

### Small-Molecule Dataset

For assessing the small-molecule conformer sampling performance of the herein-evaluated generators the Platinum Diverse Dataset^26^ was used. The dataset consists of 2859 high-quality protein-bound ligand conformations extracted from X-ray structural data provided by the PDB and has been designed and compiled especially for the benchmarking of conformer generators.^11,26,27^

### Macrocycle Dataset

To evaluate the conformational sampling of macrocycles, we used a dataset consisting of 208 macrocyclic structures taken from the Cambridge Structural Database (CSD) (130 structures),^70^ the PDB (60 structures), and the Biologically Interesting Molecule Reference Dictionary (BIRD) dataset (18 structures).^71^ The dataset (from now on referred to as the Prime dataset) was compiled by Sindhikara et al. for benchmarking the conformer generator Prime^51^ (and later was also used for benchmarking other generators^45^) and contains structurally diverse, challenging macrocyclic structures (featuring disulfide bridges, cross-linking amide bonds, and polycyclic rings, including cyclodextrins, polyglycines, cycloalkanes and peptidic macrocycles) with high crystallographic quality (low temperature factors and/or resolutions). More details about the composition of the full dataset can be found in the SI of ref (51).

### Dataset Preparation

In order to prevent any evaluated conformer generator from taking advantage of the 3D structural information present in the original dataset files, we converted both datasets into isomeric SMILES format by means of a small Python script using functionality provided by CDPKit. The thus-generated SMILES files then served as input for the conformer generators in all of the performed benchmarking runs.

### Benchmarking Software and Hardware

The benchmarking runs for each dataset were performed in a fully automated manner using a set of Bash and Python scripts on a dedicated workstation equipped with an Intel Xeon E5-2630 V3 CPU (8 × 2.4 GHz) and 64 GB of DDR3 RAM running CentOS 8 Linux. User interaction was required only for starting the conformer ensemble generation process and, once finished, for analyzing the produced conformer generator output. In each run, all evaluated conformer generators were executed in sequence without user interaction and indefinite time gaps in between. The source code of the developed benchmarking suite has been licensed under the GNU GPL V2 and is available for download (see Data and Software Availability).

### Processing Time Measurement and RMSD Calculation

Measured per-molecule processing times and total program execution times were averaged over five runs to level out runtime differences caused by background system activity and first-time program startup. Furthermore, data were read and written only from/to local hard disk drives. Thus, any influence on I/O speed and processing times resulting from changes in network latency has been excluded. Depending on availability, per-molecule processing times were either directly extracted from the saved conformer generator log files in a postprocessing step or determined indirectly by first time-stamping each line of log output generated during program execution and then calculating individual molecule processing times from the time-stamp differences of characteristic generator-specific log file lines. In order to eliminate systematic processing time errors due to internal multithreaded execution (e.g., performed in iCon), single-threaded execution was enforced for all conformer generators by tying started generator processes to a single CPU core using the Linux command “taskset”.

The RMSD of generated molecule conformers from the reference 3D structure in the processed dataset was calculated after performing an RMSD-minimizing 3D alignment using CDPKit’s implementation of the Kabsch algorithm.^68,69^ For 3D alignment and RMSD calculation, only heavy atoms were considered, and all possible topological symmetry mappings were taken into account. The lowest RMSD that could be obtained in this way among all conformers of an evaluated ensemble was then reported as the “best” RMSD. Since we encountered slight differences between the conformer ensembles generated by iCon in the five performed runs, calculated best ensemble RMSDs and ensemble sizes were averaged over the five runs for all assessed conformer generators.

### Statistical Analysis

Paired Wilcoxon signed-rank tests^72^ were carried out to check whether the observed differences between the calculated smallest reference 3D structure RMSDs of conformer ensembles generated by CONFORGE and the corresponding values obtained for the other herein-tested generators are of statistical significance (α = 0.05). The *p* values were adjusted for groupwise comparisons using the Bonferroni correction.^73^ Results of all performed tests together with a short discussion can be found in the SI.

### Assessed Generators

Aside from CONFORGE, we benchmarked four additional well-known conformer ensemble generators, Conformator, iCon, Balloon, and RDKit, and compared their conformer sampling performance with the results obtained for CONFORGE.

All assessed generators support different conformer sampling modes, methods, or parametrizations that have an impact on the size and quality of the generated conformer ensembles as well as on sampling speed. Hence, we also considered different modes of operation in the performed benchmarks: Conformator and iCon were both run with two sampling quality/speed settings (best and fast) in the small-molecule and macrocycle dataset benchmarks. For Balloon, we evaluated two sampling algorithms, and the RDKit conformer generator was assessed using its best-performing generation method in combination with two different structure refinement force fields in all performed benchmarks. In the Platinum Diverse benchmarks, CONFORGE was run in systematic sampling mode with (a) default settings for all other sampling-related parameters (“CONFORGE Systematic Default”) and (b) enabled exhaustive torsion sampling and defaults for the remaining settings (“CONFORGE Systematic Best”). For the Prime dataset benchmarks, stochastic sampling was enforced, conformer generation timeout checks were disabled, and the energy window setting was increased to 20.0 kcal/mol (default: 15 kcal/mol) to be able to generate adequate conformational ensembles also for larger macrocyclic systems in the dataset. Table 1 provides a comprehensive overview of the evaluated conformer generators, the applied settings defining each variant, and other relevant information. More in-depth details such as the specific command line executed for each tested generator variant can be found in the source code of our benchmarking suite (see Data and Software Availability).

**Table 1:** Conformer Generators and Associated Settings Employed in the Benchmarking Studies

**generator**	**settings**	**clustering**^a^	**force field**	**program version**
Balloon	DG^b^	RMSD	MMFF94	1.7.0
Balloon	GA^c^	RMSD	MMFF94	
CONFORGE	Systematic Best^d^	RMSD	MMFF94s_RTOR_NO_ESTAT^l^	CDPKit source 2023/06/28
CONFORGE	Systematic Default^e^	RMSD	MMFF94s_RTOR_NO_ESTAT^l^	
CONFORGE	Stochastic^f^	RMSD	MMFF94s_RTOR^k^	
Conformator	Best^g^	RMSD	-/MCOS^i^	1.1.0
Conformator	Fast^g^	RMSD	-/MCOS^i^	
iCon	Best^g^	RMSD	MMFF94s^j^	4.4.7
iCon	Fast^g^	RMSD	MMFF94s^j^	
RDKit	ETKDGv3^h^	–	UFF^m^	RDKit source 2020/09/2
RDKit	ETKDGv3^h^	–	MMFF94s^j^	

a Conformer similarity measure used in the compilation of diverse output ensembles.

b Genetic algorithm (GA) disabled (−noGA flag), conformers generated via DG only.

c GA enabled (default setting).

d Enforced systematic conformer sampling mode (-m systematic) with a larger energy window (20 kcal/mol), lower RMSD threshold (0.3 Å), and enabled torsion angle tolerance range sampling (-A flag).

e Enforced systematic conformer sampling mode (-m systematic) using default settings for the energy window (15 kcal/mol) and RMSD threshold (0.5 Å).

f Enforced stochastic conformer sampling (-m stochastic) using default settings for the RMSD threshold (0.5 Å), a larger energy window of 20 kcal/mol (-e 20), and conformer generation timeout checking disabled (-T 0).

g Predefined conformer generation quality levels/presets provided by the developers. For Conformator: quality level (-q option argument) 1 = Fast, 2 = Best. For iCon: presets (-t option argument) “fast” (RMSD threshold = 0.5 Å, energy window = 15 kcal/mol) and “best” (RMSD threshold = 0.8 Å, energy window = 20 kcal/mol).

h Conformer generation using DG embedding parameters for the ETKDGv3 method including improvements for small rings.^50,75^

i Macrocycle Optimization Score (MCOS), only used for macrocycle optimization.^11^

j MMFF94 parameter set variant which enforces planarity of delocalized trigonal nitrogen atoms (from now on referred to as MF in RDKit generator variant name).^76^

k MMFF94s using a refined torsion interaction parameter set.^77^

l MMFF94s_RTOR excluding electrostatic interaction terms.

m Universal Force Field (UFF, from now on referred to as UF in RDKit generator variant name).^78^

### Notes on Omega and Other Conformer Generators

The commercial conformer ensemble generator Omega^8^ by OpenEye Scientific Software, which has shown exceptional performance in several previously published benchmarks,^7,27^ would have been a perfect additional candidate for inclusion in our benchmarking studies. Unfortunately, our academic software license does not allow for publishing Omega benchmarking results without OpenEyes’s explicit permission.^74^ To enable at least a partial comparison of CONFORGE with Omega in the Platinum Diverse benchmarks, we provided the benchmarking results (see Table 2) recently published by Friedrich et al.^11^ In our studies, we used the same dataset and RMSD calculation method as Friedrich et al., and the results we obtained internally for Omega differed only insignificantly (e.g., deviations between the calculated mean accuracies were 0.012 Å for maximum ensemble size = 50 and 0.009 Å for maximum ensemble size = 250, respectively). Hardware-independent performance indicators of other commercial/open-source conformer generators (ConfGenX, ConfGen, CONFECT, MOE, Frog2, etc.) that were determined and published by Friedrich et al. for the Platinum Diverse Dataset^26,27^ can be compared with the corresponding values obtained for CONFORGE in the same way as it was done for Omega.

**Table 2:** Conformer Generator Performance Comparison for the Platinum Diverse Dataset^a^

	**maximum ensemble size 50**				**maximum ensemble size 250**			
**generator**	**mean**	**median**	**min**	**max**	**mean**	**median**	**min**	**max**
**RMSD (Å)**								
Balloon DG	0.97	0.79	0.03	4.56	0.81	0.61	0.03	4.0
Balloon GA	0.99	0.86	0.03	4.61	0.83	0.71	0.03	4.34
CONFORGE Systematic Best	**0.67**	**0.49**	0.03	3.92	**0.55**	**0.41**	0.03	3.67
CONFORGE Systematic Default	0.68	0.55	0.04	**3.14**	0.61	0.52	0.04	**2.79**
Conformator Best	0.68	0.59	**0.02**	3.26	0.57	0.47	**0.02**	2.93
Conformator Fast	0.75	0.65	**0.02**	3.51	0.64	0.54	**0.02**	3.33
iCon Best	0.70	0.59	0.03	3.66	0.64	0.57	0.03	3.66
iCon Fast	0.72	0.54	0.03	3.92	0.60	0.47	0.03	3.66
RDKit ETKDGv3 MF	0.74	0.63	0.03	3.99	0.63	0.53	0.03	3.44
RDKit ETKDGv3 UF	0.71	0.59	0.04	4.39	0.60	0.52	0.04	3.37
*Conformator Best*^b^	0.68	0.58	–	–	0.57	0.47	–	–
*Conformator Fast*^b^	0.75	0.66	–	–	0.64	0.53	–	–
*Omega*^b^	0.67	0.51	–	–	0.57	0.46	–	–
**Ensemble Size**								
Balloon DG	49	50	–	–	242	250	–	–
Balloon GA	38	43	–	–	180	210	–	–
CONFORGE Systematic Best	39	50	–	–	149	214	–	–
CONFORGE Systematic Default	29	30	–	–	83	**30**	–	–
Conformator Best	39	42	–	–	167	189	–	–
Conformator Fast	**21**	**19**	–	–	**71**	55	–	–
iCon Best	29	32	–	–	72	32	–	–
iCon Fast	35	50	–	–	122	89	–	–
RDKit ETKDGv3 MF	50	50	–	–	250	250	–	–
RDKit ETKDGv3 UF	50	50	–	–	250	250	–	–
*Conformator Best*^b^	38	42	–	–	166	187	–	–
*Conformator Fast*^b^	20	19	–	–	70	54	–	–
*Omega*^b^	34	50	–	–	118	74	–	–
**Processing Time (s)**								
Balloon DG	16.94	14.43	0.71	89.40	81.76	70.54	1.29	461.50
Balloon GA	12.60	10.98	0.04	49.99	66.59	58.27	0.47	262.12
CONFORGE Systematic Best	0.16	0.02	**0.00**	**14.3**	0.33	0.07	**0.00**	**16.08**
CONFORGE Systematic Default	**0.09**	**0.01**	**0.00**	19.79	**0.21**	**0.01**	**0.00**	19.94
Conformator Best	4.03	0.34	0.01	268.22	6.58	2.16	0.01	353.58
Conformator Fast	3.58	0.22	0.01	262.06	4.24	0.52	0.01	293.62
iCon Best	0.65	0.20	**0.00**	64.74	0.76	0.28	**0.00**	65.13
iCon Fast	0.53	0.17	**0.00**	30.98	0.65	0.27	**0.00**	31.13
RDKit ETKDGv3 MF	6.22	3.59	0.12	937.07	31.07	18.34	0.66	3682.45
RDKit ETKDGv3 UF	5.54	2.96	0.10	932.94	27.38	14.90	0.54	3657.46

a The best values for RMSD, ensemble size, and molecule processing time obtained by any assessed generator are written in bold letters.

b The values listed for Conformator were taken from ref (11) and are included for reference to demonstrate the correctness of our benchmarking code by the close reproduction of the previously published results for this generator. As a side effect, this allows us to also include the results published in ref (11) for Omega, which we could not assess in our study due to licensing reasons.

### Small-Molecule Conformer Sampling Performance

As shown in Table 2, our new conformer generator CONFORGE is able to retrieve the bioactive conformations for the input molecules more accurately on average than all of our competitors.

For the tested maximum ensemble sizes of 50 and 250, the improvement of “CONFORGE Systematic Best” over all competitors is of high statistical significance (α = 0.05; Tables S1 and S3). This is achieved while beating them regarding runtime, most methods even by more than an order of magnitude. Conformator is able to find better conformations when only regarding the best conformations from their produced ensembles, but as the baseline conformation would not be available in practical applications, these better conformations would not be reliably retrievable. Conformator also produces smaller ensemble sizes when run with its “Fast” presets, which may be generally desirable, but as the user of a given method will have to continue working with the generated conformations, the on-average worse results are a significant downside. The performance benefits of CONFORGE over Conformator are also large enough to have real-world impact with a speedup of 19× (see Table 3) on average with a maximum ensemble size of 50 when comparing CONFORGE’s “Best” to Conformator’s “Fast” variant, which is the worst-case comparison for our method. The runtimes of our benchmarks also show that no other openly available method can compete with our new implementation regarding time spent, with the best competitor, the commercially available iCon conformer generator with “Fast” presets, still requiring 2.9× the processing time of our method (see Table 3).

**Table 3:** Total Program Execution Times and Molecule Processing Failures recorded for the Platinum Diverse Dataset

	**maximum ensemble size 50**		**maximum ensemble size 250**	
**generator**	**total execution time(hh:mm:ss)**^a^	**number of failed molecules**	**total execution time(hh:mm:ss)**^a^	**number of failed molecules**
Balloon DG	13:26:45	0	64:55:43	1
Balloon GA	10:18:53	3	53:12:55	3
CONFORGE Systematic Best	00:08:56	0	00:20:06	0
CONFORGE Systematic Default	00:05:21	0	00:12:49	0
Conformator Best	03:11:41	4	05:13:06	4
Conformator Fast	02:50:25	4	03:21:48	4
iCon Best	00:31:59	0	00:37:46	0
iCon Fast	00:26:07	0	00:33:44	0
RDKit ETKDGv3 MF	05:09:01	1	25:44:29	1
RDKit ETKDGv3 UF	04:36:43	1	22:48:01	1

a Wall time elapsed between program start and termination.

It is also important to note that most other conformer generators were not able to handle all of the presented molecules and failed to produce any results for some molecules. The overall program execution times and numbers of molecules for which processing failed are listed in Table 3. Only iCon was also able to compute conformers for all molecules regardless of ensemble size, while again, all competitors were significantly slower than our method. The computation of the conformers for the whole dataset took the more thorough “CONFORGE Systematic Best” variant under 9 min, while the commercially available iCon took 26 min with the “Fast” variant. Freely available algorithms required at least 2 h 50 min (“Conformator Fast”), with many of them taking significantly longer than even that method. This comparison nicely visualizes the real impact of these runtimes on a researcher whose work depends on the generation of conformers.

As can be seen in Table 4, the largest improvement of CONFORGE regarding accuracy can be identified in the number of molecules for which conformer generation produces especially similar results to the true bioactive conformation (RMSD < 0.5 Å). “CONFORGE Systematic Best” is thereby able to produce more conformers close to this ground truth while still performing similar to the competitors for larger RMSD accuracy ranges.

**Table 4:** Percentiles of Platinum Diverse Dataset Structures Successfully Reproduced below Specified RMSD Thresholds (0.5–2.0)^a^

	**maximum ensemble size 50**				**maximum ensemble size 250**			
**generator**	**0.5**	**1.0**	**1.5**	**2.0**	**0.5**	**1.0**	**1.5**	**2.0**
Balloon DG	32.4	61.1	78.5	89.6	41.8	69.7	84.5	93.6
Balloon GA	26.6	58.2	79.2	91.1	34.2	67.6	86.6	94.7
CONFORGE Systematic Best	**51.6**	79.2	90.6	96.4	**59.7**	**86.8**	95.2	98.4
CONFORGE Systematic Default	44.6	80.5	92.7	97.3	47.5	85.8	95.8	99.0
Conformator Best	41.8	78.0	**93.2**	**98.4**	52.9	85.9	**96.5**	99.0
Conformator Fast	36.8	73.8	91.8	98.0	45.9	83.2	95.5	98.8
iCon Best	36.5	**80.8**	93.1	97.9	38.0	86.6	96.2	99.0
iCon Fast	46.1	76.5	89.5	96.5	54.3	85.3	95.1	98.4
RDKit ETKDGv3 MF	39.8	73.8	91.2	98.0	46.7	83.6	96.0	99.2
RDKit ETKDGv3 UF	40.9	75.7	92.0	97.8	47.6	84.9	96.5	**99.4**
*Conformator Best*^b^	42	78	94	98	53	86	97	99
*Conformator Fast*^b^	37	73	91	98	46	83	95	99
*Omega*^b^	49	80	92	97	56	87	96	99

a The best value for each RMSD threshold obtained by any assessed generator is written in bold letters.

b The values listed for Conformator were taken from ref (11) and are included for reference to demonstrate the correctness of our benchmarking code by the close reproduction of the previously published results for this generator. As a side effect, this allows us to also include the results published in ref (11) for Omega, which we could not assess in our study due to licensing reasons.

**Figure 10:**
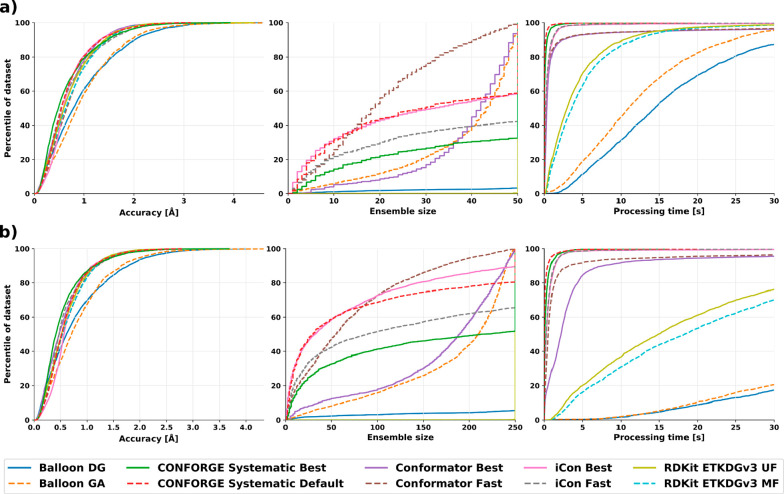
Percentile of Platinum Diverse Dataset molecules as a function of accuracy in the reproduction of the protein-bound ligand conformation (left), output ensemble size (middle), and molecule processing time (right) at maximum ensemble sizes of (a) 50 and (b) 250 conformers. For each of the diagrams, faster-rising curves indicate better performance.

In practice, the ensemble size produced during conformer generation is also an important factor. If a large number of conformers are produced, it is more likely that one of them is close to the bioactive conformation, as a fundamental goal of conformer generation is to produce a sufficiently diverse set of results. Still, this increase in the number of conformations comes with the downside of having to search through this larger solution space for the conformer(s) desirable for a given use case and results in a larger uncertainty about whether the correct one was chosen. For this reason, we visualized in Figure 10 how the accuracy, ensemble size, and processing times compare between the different conformer generation methods. We limited the ensemble sizes to 50 and 250 for the different measurements, and it can be seen that CONFORGE lies approximately in the middle regarding how much of the ensemble size limit it uses, with the “Best” variant producing smaller ensembles than the “Default” variant. Generally, all methods would be allowed to output maximally large ensemble sizes (exactly the maximum allowed number for each molecule), but with the exception of RDKit, they all discard too similar solutions. Even though CONFORGE produces similarly sized ensembles, its runtime is unparalleled by the other methods, and the accuracy is nearly always better. This shows that the aforementioned benefits in accuracy and, especially, runtime are not an artifact of larger ensembles.

**Figure 11:**
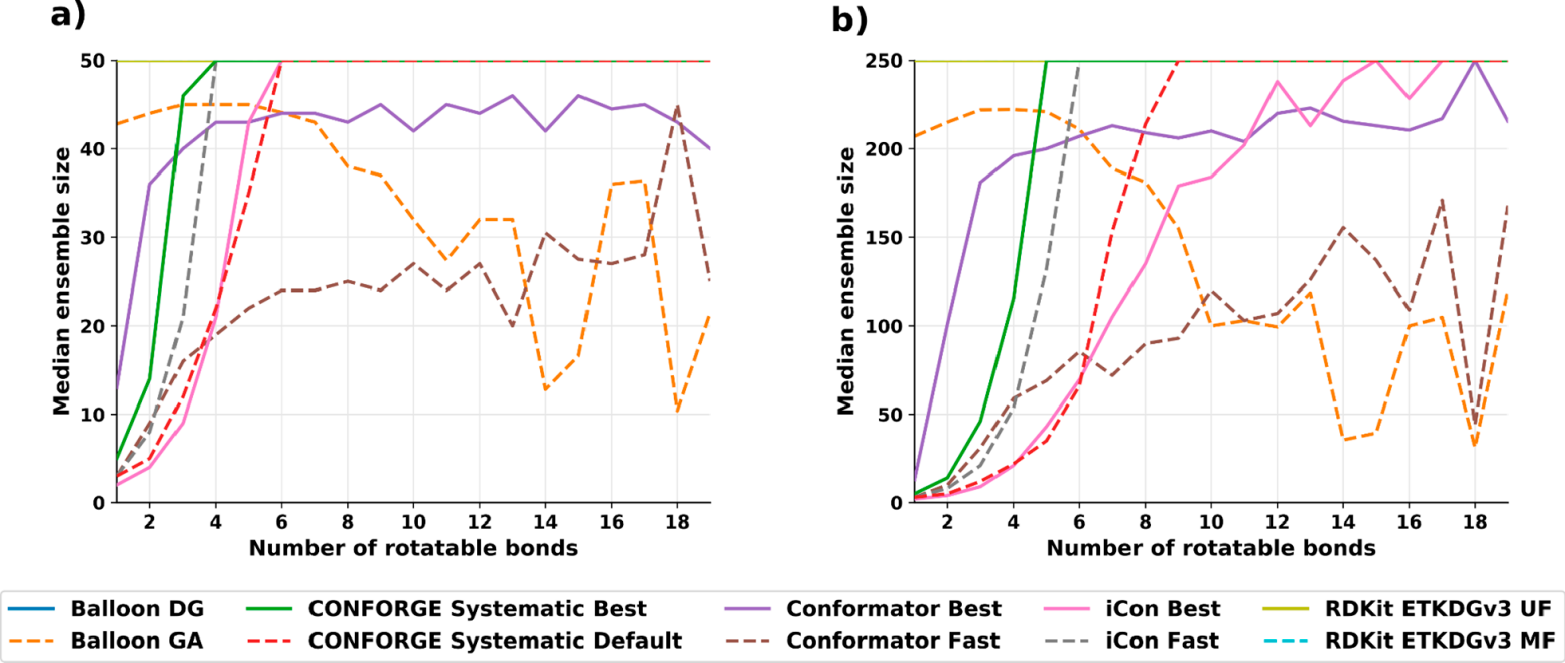
Median ensemble size as a function of the number of rotatable bonds at maximum ensembles of (a) 50 and (b) 250 conformers generated for the Platinum Diverse Dataset. Curves with lower median ensemble sizes indicate better performance with respect to this criterion.

The size of the ensembles usually relates to the degrees of freedom that can be sampled during conformer generation. Rotatable bonds are thereby the largest contributing factor, as the 3D position of molecule substructures connected to each of these bonds can vary, leading to a combinatorial problem when assembling the final conformer. Figure 11 shows this property nicely, as most conformer generators produce larger ensembles with an increasing number of rotatable bonds. Exceptions to this are the variants of Balloon, which seem to be agnostic to this number, and Conformator’s “Best” variant, which does not use the full amount of allowed conformers, even for complex molecules.

**Figure 12:**
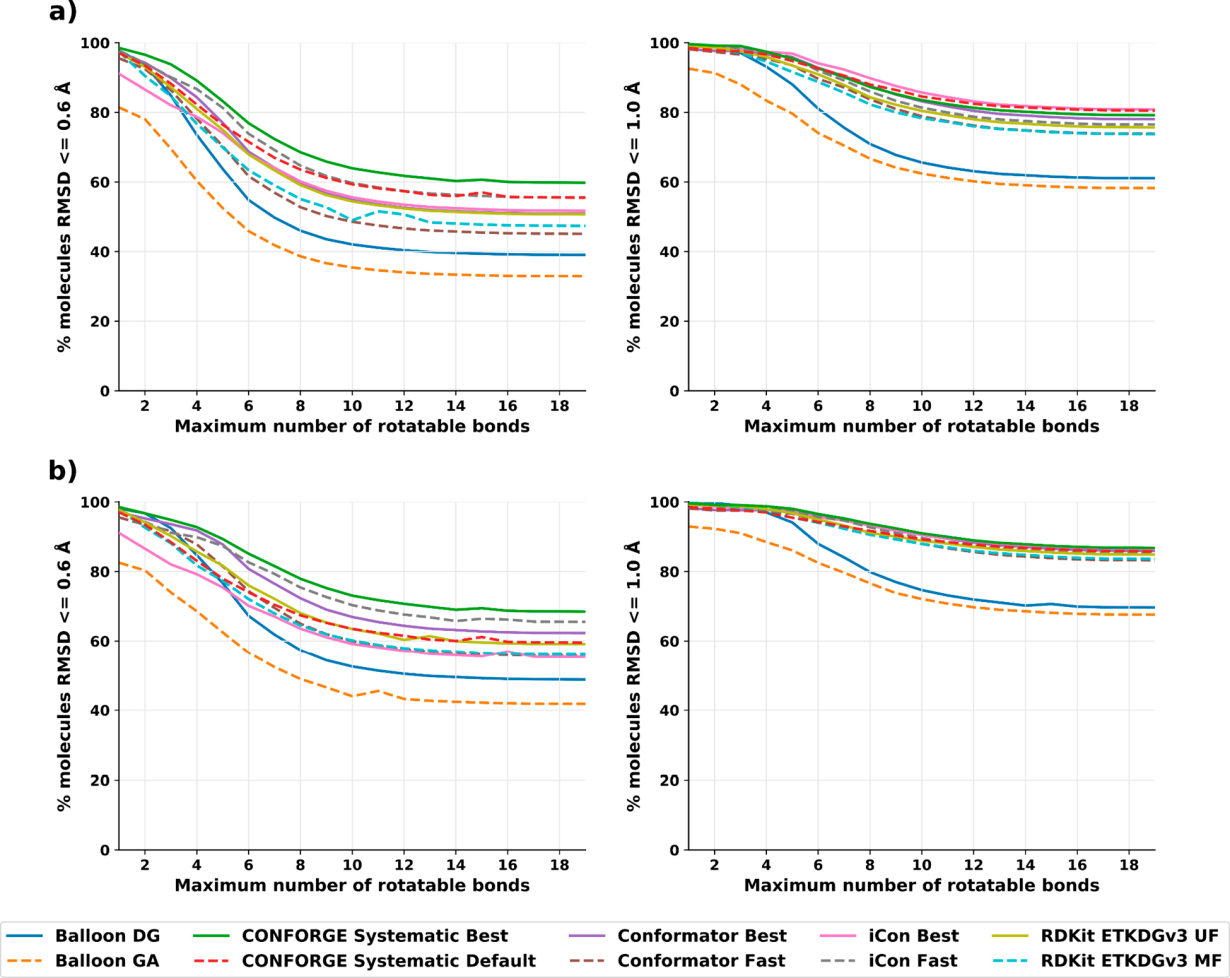
Percentile of Platinum Diverse Dataset molecules reproduced by the assessed generators with a maximum RMSD of 0.6 (left) and 1.0 Å (right) as a function of the maximum number of rotatable bonds at maximum ensemble sizes of (a) 50 and (b) 250 conformers.

The molecules are also likely to contain more atoms with an increasing number of rotatable bonds. This introduces more possibilities of errors in the relative position of the atoms compared to the crystal structure, leading to a higher expected RMSD than for small molecules when using the same conformer generation method and therefore lower accuracy. With increasing molecule size, this inevitably leads to a decreasing number of molecules retrieved with any fixed RMSD precision. Figure 12 visualizes this relationship for the different benchmarked methods and shows how the different algorithms are affected by this phenomenon. While all methods retrieve fewer molecules within a fixed threshold with an increasing number of rotatable bonds, the CONFORGE variants are usually able to handle this increase better than the competitors. Not only is the retrieval of molecules with few rotatable bonds more accurate, but larger ones with more rotatable bonds also experience less of an accuracy loss compared to the other benchmarked methods. Other methods we could identify to handle larger numbers of rotatable bonds well include “iCon Best” (1 Å), “Conformator Best” (1.5–2 Å), and “RDKit ETKDGv3 UF” (2 Å), which, as can be seen in Table 4, show good reproduction rates with increasing RMSD thresholds. For the percentage of molecules retrieved within the accuracy threshold of RMSD ≤ 0.5 Å. “CONFORGE Systematic Best” outperformed all other methods.

### Macrocycle Conformer Sampling Performance

Macrocyclic compounds usually pose an even larger challenge for conformer generators due to the large number of (partially) rotatable bonds contributed by flexible side chains and the macrocyclic ring system itself. For this reason, we separately compare the performance of the different conformer generation methods to our “CONFORGE Stochastic” variant, which has been specifically designed for macrocycles and large/complex compounds.

Table 5 shows the performed measurements. “CONFORGE Stochastic” achieves on average higher accuracy than most other herein-benchmarked methods, with high statistical significance (α = 0.05, Table S5). The only method for which we could not reject the null hypothesis, that the RMSD results follow the same random distribution, was “RDKit ETKDGv3 MF”. When the accuracy was only measured for atoms constituting the macrocyclic ring, it can be observed that the relative improvement over other methods is even larger. Still, this improved conformer quality comes at the cost of longer runtimes compared with other methods. As the average quality of our results is better and the relevance of macrocyclic compounds for drug development is continuously rising, our new method will provide drastic improvements when working with such molecules. The only notable competitor in this domain is the RDKit variant “ETKDGv3 MF”. This method is able to outperform “CONFORGE Stochastic” regarding runtime while producing conformers that closely resemble experimental 3D structures.

**Table 5:** Conformer Generator Performance Comparison for the Prime Macrocycle Dataset^a^

**generator**	**mean**	**median**	**min**	**max**
**Overall RMSD (Å)**^b^				
Balloon DG	1.78	1.43	0.04	7.64
Balloon GA	1.85	1.59	**0.03**	5.83
CONFORGE Stochastic	**1.11**	**0.88**	0.04	5.04
Conformator Best	1.56	1.19	0.18	6.48
Conformator Fast	1.62	1.25	0.22	6.47
iCon Best	1.47	1.12	0.05	6.37
iCon Fast	1.48	1.17	0.05	5.93
RDKit ETKDGv3 MF	1.17	**0.88**	**0.03**	**4.71**
RDKit ETKDGv3 UF	1.25	0.98	0.05	4.84
**Macrocycle RMSD (Å)**^c^				
Balloon DG	0.94	0.74	0.03	3.86
Balloon GA	1.14	0.92	0.03	4.53
CONFORGE Stochastic	**0.56**	**0.46**	0.03	**2.36**
Conformator Best	0.97	0.81	0.13	5.19
Conformator Fast	1.03	0.86	0.13	3.84
iCon Best	0.91	0.71	0.04	4.75
iCon Fast	0.95	0.76	0.04	5.24
RDKit ETKDGv3 MF	0.62	0.49	**0.02**	3.26
RDKit ETKDGv3 UF	0.67	0.58	0.03	3.14
**Ensemble Size**				
Balloon DG	321	500	–	–
Balloon GA	145	64	–	–
CONFORGE Stochastic	342	390	–	–
Conformator Best	236	233	–	–
Conformator Fast	100	59	–	–
iCon Best	**69**	**44**	–	–
iCon Fast	131	69	–	–
RDKit ETKDGv3 MF	500	500	–	–
RDKit ETKDGv3 UF	500	500	–	–
**Processing Time (s)**				
Balloon DG	262.62	174.45	9.87	1,453.09
Balloon GA	494.88	343.06	40.58	2,602.07
CONFORGE Stochastic	1,005.98	426.73	16.04	12,485.50
Conformator Best	471.40	169.61	7.19	43,358.54
Conformator Fast	186.90	111.53	1.54	5,615.60
iCon Best	28.53	30.17	0.72	**492.15**
iCon Fast	**26.05**	**24.91**	**0.68**	574.71
RDKit ETKDGv3 MF	703.26	178.30	8.59	35,138.18
RDKit ETKDGv3 UF	677.82	159.89	7.25	34,714.72

a The best values for RMSD, ensemble size, and molecule processing time obtained by any assessed generator are written in bold letters.

b RMSD taking into account all heavy atoms of the molecule.

c RMSD taking into account only the heavy atoms constituting the macrocyclic ring system.

For the sake of a fair comparison of the results, it has to be again noted that—in contrast to all other assessed generators—the output conformers produced by the RDKit generator variants (for technical reasons) have not been postprocessed and were not constrained in any way regarding their energy and mutual structural similarity. At the expense of output ensemble size, this not only significantly reduces the per-molecule processing time but also avoids the risk of losing generated conformers closely resembling experimental 3D structures of interest (such as the bioactive conformation used as the reference for accuracy calculations).

The difficulty of this task can be seen once again in Table 6, where the number of molecules for which the conformer generation failed is shown. Besides “CONFORGE Stochastic”, only RDKit was able to complete the computations without failure, while the other methods could not produce any results for at least one molecule. Notably, Conformator even completely crashed with a segmentation fault when presented six of the molecules in the dataset (VEVHAF, POTTEY, PRD_000785, 1YND_SFA, 1NMK_SFM, and 3I6O_GR6).

**Table 6:** Total Program Execution Times and Molecule Processing Failures Recorded for the Prime Macrocycle Dataset

**generator**	**total execution time(hh:mm:ss)**^a^	**number of failed molecules**
Balloon DG	14:31:35	9
Balloon GA	27:20:21	11
CONFORGE Stochastic	58:08:40	0
Conformator Best	26:29:35	7
Conformator Fast	10:46:42	9
iCon Best	01:48:45	1
iCon Fast	01:40:27	1
RDKit ETKDGv3 MF	40:37:57	0
RDKit ETKDGv3 UF	39:09:47	0

a Wall time elapsed between program start and termination.

Just as for the previous benchmark, we computed the percentiles of molecules for which conformers within a certain RMSD threshold have been found by the individual methods. Table 7 shows the resulting values, with “CONFORGE Stochastic” finding the largest number of high-quality conformers for any of the presented thresholds. The next best percentile values were measured for “RDKit ETKDGv3 MF”.

**Table 7:** Percentiles of Prime Macrocycle Dataset Structures Successfully Reproduced below Specified RMSD Thresholds (0.5–4.0)^a^

	**RMSD threshold (Å)**							
**generator**	**0.5**	**1.0**	**1.5**	**2.0**	**2.5**	**3.0**	**3.5**	**4.0**
Balloon DG	16.3	35.6	49.0	61.1	70.7	77.9	82.7	87.5
Balloon GA	5.8	23.6	43.8	59.6	71.6	79.3	85.6	90.4
CONFORGE Stochastic	**27.9**	**55.8**	**74.5**	**83.7**	**92.8**	**96.6**	**98.1**	**99.0**
Conformator Best	11.5	39.9	60.6	70.7	80.3	86.5	89.4	92.3
Conformator Fast	7.2	36.1	55.3	69.2	78.4	84.6	88.9	90.9
iCon Best	19.2	45.7	60.6	72.6	80.8	90.4	93.7	95.7
iCon Fast	25.0	44.7	60.6	70.7	80.8	88.0	93.8	95.7
RDKit ETKDGv3 MF	26.0	54.8	70.2	80.3	89.9	95.2	98.1	**99.0**
RDKit ETKDGv3 UF	22.6	51.4	67.8	79.8	89.4	94.7	97.6	98.6

a The best value for each RMSD threshold obtained by any of the assessed generators is written in bold letters.

**Figure 13:**
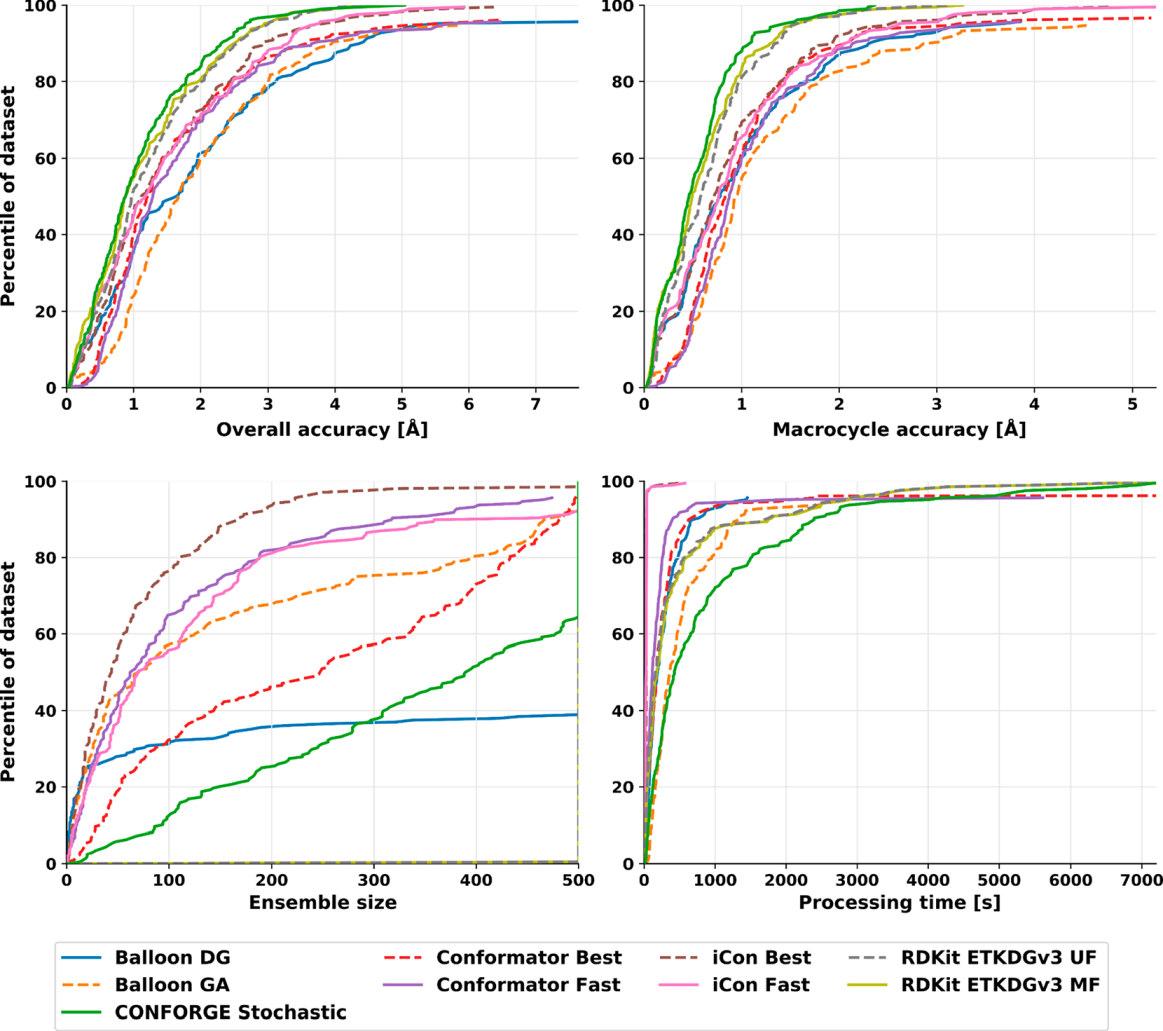
Percentile of Prime Macrocycle Dataset molecules as a function of accuracy in the reproduction of the crystallographic 3D structure (left), output ensemble size (middle), and molecule processing time (right). For each of the diagrams, quicker-rising curves indicate better performance.

The accuracy benefits of CONFORGE can, once again, be seen in Figure 13. The produced ensembles are of average size compared with our competitors, while the method needs more time to produce the results than others. This increase in computation time should easily be made up by the increase in quality over the other algorithms.

**Figure 14:**
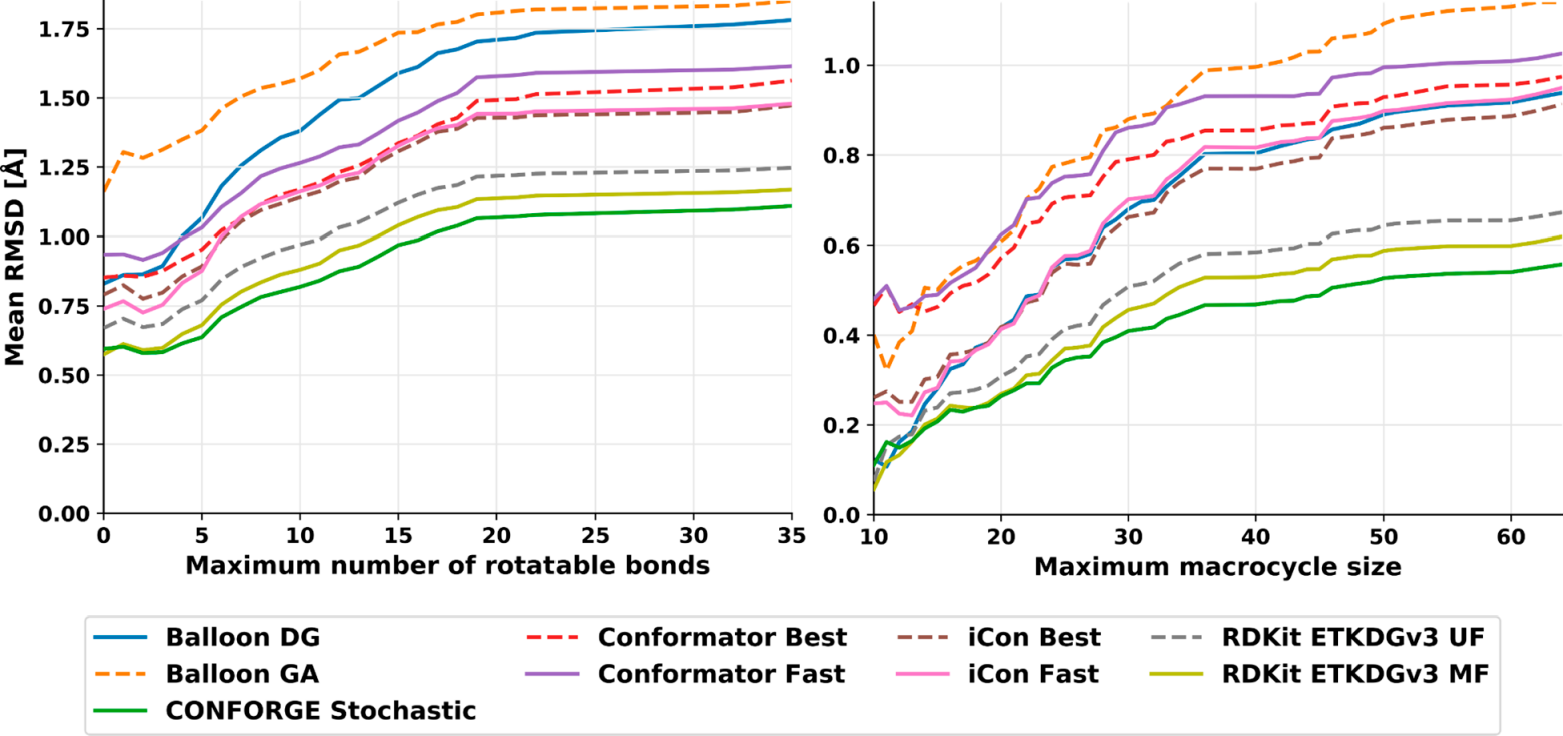
Mean overall RMSD as a function of the maximum number of rotatable side chain bonds (left) and maximum size (atom count) of the macrocyclic ring system (right) for the Prime Macrocycle Dataset. Curves with a lower mean RMSD indicate better performance with respect to this criterion.

The quality of CONFORGE’s results is visualized cumulatively over the number of rotatable bonds and the size of the macrocycle in Figure 14. There, the plots show that for nearly all possible thresholds for those two properties, our method outperforms any other algorithm regarding the accuracy of generated conformers.

## Conclusions

We have introduced and described the implementation of the novel conformer ensemble generator CONFORGE, which has been designed and developed to deliver top-level performance for all types of organic molecules in the drug-like chemical space. CONFORGE is fully open-source and available as part of the Chemical Data Processing Toolkit in the form of a versatile command-line tool that accepts a wide panel of input data formats and a set of classes and functions provided by CDPKit’s C++/Python-API. CONFORGE’s implementation is based on established concepts and algorithms that have proven their power with respect to conformer ensemble generation in other well-known software tools for this purpose. For the computationally efficient and accurate conformational sampling of drug-like small molecules, a knowledge-based systematic approach is employed, which makes extensive use of pregenerated fragment and torsion angle libraries that were derived from experimental 3D structures. For the sampling of macrocycle conformers, CONFORGE implements a purely stochastic approach based on a combination of DG and 3D structure refinement by an iterative MMFF94 energy minimization. Irrespective of the sampling approach, CONFORGE does not require any input atom 3D coordinates and is able to generate conformer ensembles solely from molecular graph connection table information. The conformer sampling approach best suited for a processed input molecule is either chosen automatically or can be specified by the user in advance for all molecules to process. Furthermore, CONFORGE correctly handles compounds consisting of multiple molecules such as salts and mixtures by a separate generation and later combination of individual component conformer ensembles.

CONFORGE’s capability to reproduce experimental 3D structures and its computational efficiency and robustness have been assessed for typical drug-like organic molecules using the Platinum Diverse Dataset and for macrocyclic systems using a dataset of 208 molecules compiled by Sindhikara et al.^51^ The calculation of performance metrics and the visual presentation of the obtained results largely followed the established protocol developed by Friedrich et al.^26^ with extensions for the presentation of macrocycle sampling results. For comparison, several well-known commercial (iCon, two modes), non-open-source (Conformator, two modes), and open-source conformer generators (Balloon, two modes; RDKit, two variants) were benchmarked in addition to CONFORGE using the same benchmarking protocol. For the Platinum Diverse Dataset benchmarks, two runs with maximum output ensemble sizes (MESs) of 50 and 250 representative conformers were performed, and for testing of the macrocycle sampling performance, a MES of 500 was chosen. In the Platinum Diverse Dataset benchmarks, CONFORGE achieved median accuracies in the reproduction of bioactive conformations of 0.49 Å (MES = 50) and 0.41 Å (MES = 250), which were the best values among all tested generators (see Table 2). At the same time, CONFORGE had the lowest median processing time per molecule (12 ms for MES = 50 and 14 ms for MES = 250) and the lowest mean output ensemble size of 29 conformers in the case of a MES of 250 and the second lowest for a MES of 50, respectively. The mean accuracy of 1.11 Å achieved by CONFORGE for the macrocycle dataset was again the best among all benchmarked conformer ensemble generators (see Table 5). However, CONFORGE also showed the highest median per-molecule processing time (426.73 s) and produced output ensembles with a median size of 390 conformers (Table 5). These results indicate that there is still room for improvements when it comes to macrocycle conformer sampling and that CONFORGE will be best suited for low-throughput applications which favor accuracy over speed.

In summary, the presented open-source conformer ensemble generator CONFORGE has proven to be able to deliver excellent performance in several aspects of relevance. To our knowledge, CONFORGE is the first open-source conformer generator that is able to compete with leading commercial software for the conformational sampling of drug-like small molecules and at the same time also shows very good performance when it comes to the conformational sampling of macrocyclic systems. It will provide the scientific community with a truly free alternative of high quality that facilitates open research due to the absence of any restrictions on the use of the generated data. As part of the CDPKit project, CONFORGE will be actively maintained and undergo further development. Planned future improvements of CONFORGE include speed optimizations regarding stochastic sampling, the possibility to use other general-purpose force fields like the Open Force Field,^79^ and functionality enabling a restriction of conformer sampling to only particular user-specified parts of the processed molecule.

## Data and Software Availability

The source code of the developed benchmarking suite, both test datasets in SDF and SMILES format, the generated conformer ensembles (multi-conformer SD files) and benchmarking result files (CSV files and figures) for all assessed generators, the CDPKit source code in the version at the time of benchmarking, and an installer for the corresponding CDPKit binaries (compiled for RHEL 8.x-based systems) can be downloaded from https://zenodo.org/record/8137603 (DOI: 10.5281/zenodo.8137603).

## Supporting Information Available

Additional methodological sections Molecule Fragmentation, Fragment Conformer Ensemble Generation, Fragment Conformer Postprocessing, and Multimolecule Output Conformer Ensemble Generation; Figures S1–S3; Tables S1–S5 plus accompanying text summarizing the results of the carried-out paired Wilcoxon sign rank tests; Table S6 listing all options supported by the CONFORGE command-line interface (PDF)
