# Supplementary material for: High-Quality Conformer Generation with CONFORGE: Algorithm and Performance Assessment
Source: J Chem Inf Model. 2023 Aug 25;63(17):5549–70. doi: 10.1021/acs.jcim.3c00563 (PMC10498443; doi:10.1021/acs.jcim.3c00563)
Supplement: Supplementary file 1 — ci3c00563_si_001.pdf [file ci3c00563_si_001.pdf]

Supplementary Information

High-Quality Conformer Generation with CONFORGE: Algorithm and Performance Assessment

Thomas Seidel[a*], Christian Permann[b], Oliver Wieder[c], Stefan M. Kohlbacher[a] and Thierry Langer[abc]

[a]Department of Pharmaceutical Sciences, Division of Pharmaceutical Chemistry, University of Vienna, Josef-Holaubek-Platz 2, 1090 Vienna, Austria
[b]NeGeMac Research Platform, Department of Pharmaceutical Sciences, Division of Pharmaceutical Chemistry, University of Vienna, Josef-Holaubek-Platz 2, 1090 Vienna, Austria
[c]Christian Doppler Laboratory for Molecular Informatics in the Biosciences, Department of Pharmaceutical Sciences, Division of Pharmaceutical Chemistry, University of Vienna, Josef-Holaubek-Platz 2, 1090 Vienna, Austria

* Corresponding author
e-mail: thomas.seidel@univie.ac.at

Molecule Fragmentation

As described in section Systematic Conformer Sampling of the main manuscript, the systematic conformer sampling procedure generates molecule conformers by assembling selected pre-computed conformers of molecular fragments. These fragments are derived from the molecular graph by cutting specific carbon-heteroatom bonds and bonds to ring system substituents. The pseudo-code function `isCutBond()` shown in Listing S1 implements the rules by which the bonds being cut are identified:

```
bool isCutBond(Bond b):
  if isInRing(b) or connectsHydrogenAtom(b):
    return false

  if degree(b.atom1) < 2 or degree(b.atom2) < 2:
    return false

  if isInRing(b.atom1) or isInRing(b.atom2):
    return true

  if order(b) != 1:
    return false

  if element(b.atom1) == 'C':
    if element(b.atom2) not in { 'N', 'O', 'S', 'P', 'Se' }:
      return false
  elif element(b.atom2) == 'C':
    if element(b.atom1) not in { 'N', 'O', 'S', 'P', 'Se' }:
      return false
  else:
    return false

  if hasNonSingleBondTo(b.atom1, { 'N', 'O', 'S' }) or
     hasNonSingleBondTo(b.atom2, { 'N', 'O', 'S' }):
    return false

  return true
```

Listing S1. Pseudo-code implementing rules for the identification of bonds to cut in the molecule fragmentation process.

In the resulting fragments, bonds to previously connected fragments of the parent molecule are preserved. Their atoms are replaced by corresponding pseudo atoms encoding chemical element, hybridization state, formal charge and membership in aromatic rings. These pseudo atoms provide enough local structural context for a later library lookup/on-the-fly generation of proper three-dimensional (3D) fragment structures. Figure S1 shows an example of the fragmentation of a typical drug-like organic molecule and the list of resulting molecular fragments obtained by cutting bonds that were identified with the mentioned rules.

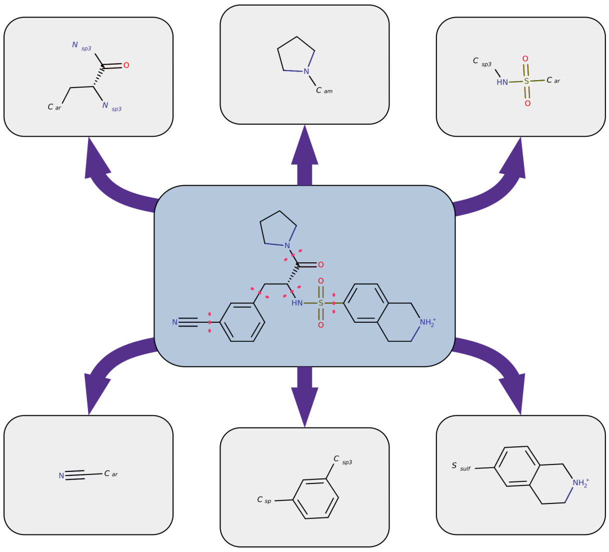

Figure S1. Example for the generation of fragments by splitting specific bonds of a molecule (marked by dashed red lines). Information about the type and nature of formerly connected atoms is carried over to the resulting fragments by the introduction of pseudo atoms which encode chemical element, hybridization state, formal charge and membership in aromatic rings.

Fragment Conformer Ensemble Generation

Depending on specified settings, data availability, and structural type, fragment conformer ensembles are either derived from provided input 3D coordinates, retrieved/derived from an entry in the built-in or user-specified fragment library, taken from the runtime cache, or have to be generated from scratch.

Figure S2 outlines the conformer ensemble generation workflow for a given input fragment. The workflow starts with checking whether the conformers should (by default, input coordinates are discarded) and can be derived from the provided atom 3D coordinates. Supplied atom 3D coordinates are only considered if they are present for at least all heavy atoms of the fragment, and if the fragment is not a flexible ring system or ring conformer enumeration has been disabled. If so, all present 3D coordinates are extracted and a calculation of missing hydrogen coordinates is performed (the hydrogen 3D coordinates calculation procedure is briefly described in the main manusript section Random Conformer Generation). Afterwards, the resulting complete fragment 3D structure is forwarded to the conformer post-processing step (see supplementary section Fragment Conformer Post-Processing).

If input coordinates should not or cannot be considered according to the initial checks, the workflow proceeds with the fragment canonicalization step that comprises three sub-steps: In the first step, terminal heavy atoms connected to atoms of aromatic rings are replaced by hydrogen. Fragments differing only in their aromatic ring system substitution pattern thus converge into the same canonical fragment structure. This measure helps to increase the

fragment library/runtime cache hit rate for quite common aromatic fragments like substituted phenyl rings. The resulting incorrect lengths of bonds between aromatic ring and replaced substituent atoms are corrected later in the conformer post-processing step (see supplementary section Fragment Conformer Post-Processing).

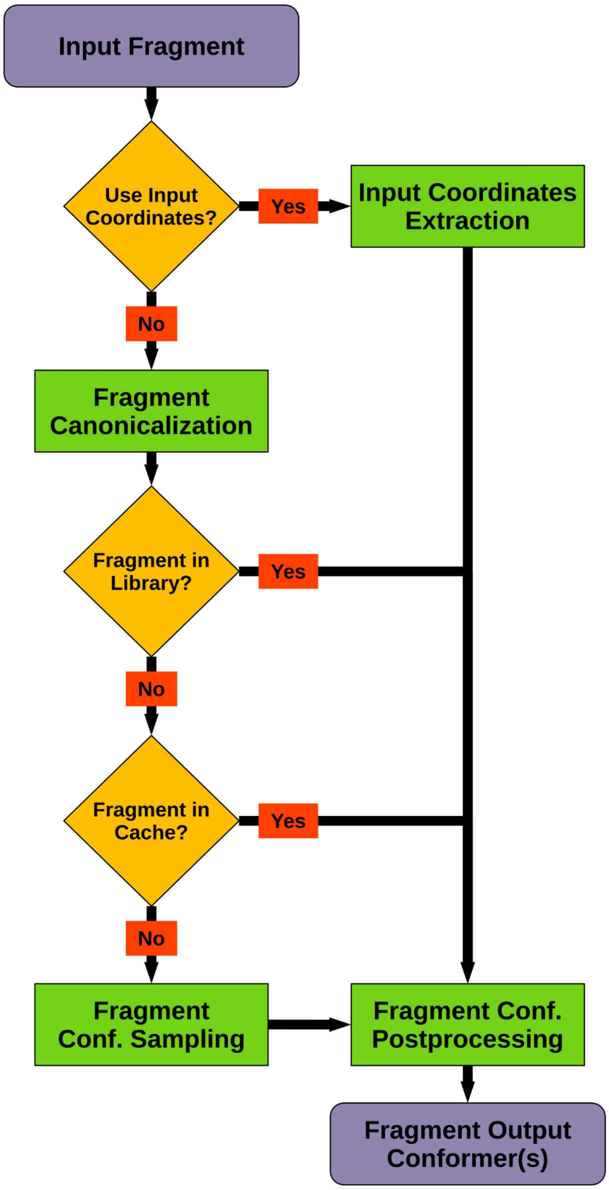

Figure S2. Fragment conformer ensemble generation workflow. Further details on the fragment conformer post-processing step can be found in supplementary Figure S3.

In the next step, free valences of pseudo atoms (see supplementary section Molecule Fragmentation) are compensated by adding explicit hydrogens and a calculation of canonical atom labels using CDPKit's implementation of the McKay algorithm[1] is performed. Finally, a binary representation of the fragment's connection table with atom and bond lists ordered according to the previously calculated canonical atom labels is generated. The

calculated SHA1 hash code[2] of the connection table data is then converted to a 64 bit integer key which serves as a unique fragment identifier (ID) in subsequent processing steps.
In the canonical atom labelling and fragment ID calculation procedure, stereodescriptors of defined stereocenters are only considered if the processed fragment represents a ring system. Stereochemistry is disregarded for acyclic fragments since configurations of tetrahedral stereocenters and double bonds can be corrected easily by fast geometric operations in the conformer post-processing step (see supplementary section Fragment Conformer Post-Processing). Furthermore, for each acyclic fragment only a single random 3D structure is stored in the fragment library and runtime cache in order to reduce per-fragment memory consumption and thus allow for a higher number of entries. Complete conformer ensembles are then generated in the post-processing step by performing torsion driving on the saved 3D structure. The increased post-processing effort resulting from these measures is condoned to attain higher library and runtime cache hit rates for this structurally highly diverse fragment type. High hit rates are key for achieving low average molecule processing times by avoiding the relatively slow distance geometry-based random conformer generation procedure[3,4] for as many encountered fragments as possible.
After the fragment canonicalization procedure, the calculated 64 bit fragment ID is used as a unique key for querying the loaded fragment libraries. Aside from the built-in fragment library which gets loaded on program startup, CONFORGE also supports multiple user-specified external fragment libraries which are searched one by one for an entry matching the supplied fragment ID. If one of the performed library lookups is successful, the fragment conformers deposited in the library entry are extracted and forwarded to the final post-processing step after which the workflow terminates.
Should all library lookups fail, an attempt is made to find a matching entry in the dynamic runtime cache. The runtime cache is implemented as a Least Recently Used (LRU) list (limited to 10,000 entries in the current implementation) and stores conformers of previously processed fragments that likewise could not be found in any of the searched libraries. An identified matching cache entry is then processed in the same way as a corresponding library hit.
If library and cache lookups both fail, the requested fragment conformers are generated from scratch based on the information provided by the molecular graph of the derived canonical fragment. 3D structures are generated by means of the previously described random conformer generation functionality (see main manuscript section Random Conformer Generation). For acyclic fragments and fragments representing purely aromatic ring systems, just a single output 3D structure is generated. Conformers of fragments representing flexible ring systems are sampled stochastically using a procedure similar to the one described in section Stochastic Conformer Sampling of the main manuscript. Depending on actual ring system flexibility, this usually leads to the output of multiple structurally diverse (default ring atom root-mean-square deviation (RMSD) = 0.1 Å) low-energy 3D structures which cover the conformational space of the fragment in the effective energy window (default values: small ring systems 8 kcal/mol, macrocycles 25 kcal/mol). Afterwards, the generated 3D structures are deposited in the runtime cache for potential later reuse and then get post-processed in the workflow's final step.

Fragment Conformer Post-Processing

Fragment conformers resulting from the procedure described in the previous section need to undergo further checks, corrections and modifications before they can be used as building blocks for molecule 3D structure templates (see main manuscript section Systematic Conformer Sampling). Depending on fragment type and source of the input conformers, required post-processing steps comprise: Correction of aromatic ring substituent bond lengths, inversion of stereocenters for the correction of detected configuration errors, enumeration of invertible nitrogen states and generation of additional conformers by torsion driving. The workflow of the executed post-processing procedure is shown in Figure S3. Herein, the first two processing steps are concerned with required structural corrections resulting from molecular graph modifications and the disregard of stereochemistry for acyclic fragments in the fragment canonicalization procedure (see previous section).

In the first correction step, for every input conformer, the lengths of bonds between aromatic ring atoms and atoms of exocyclic substituents (which were replaced by hydrogen in the fragment canonicalization procedure, see previous section) get scaled to match the corresponding MMFF94[5] equilibrium bond lengths in the structural context of the parent molecule. In the second step, atom and bond stereocenters of acyclic fragments whose calculated configuration does not match the desired output configuration are corrected by exchanging stereocenter substituent positions by means of geometric transformations applied on the involved atom 3D coordinates.

Since systematic errors introduced in the fragment canonicalization procedure do not apply to fragment conformers extracted from a supplied 3D structure of the parent molecule (see Figure S3), the two correction steps can be bypassed for this sort of conformers which is realized by a dedicated check at the beginning of the post-processing workflow.

The next processing step performs a systematic enumeration of all possible invertible nitrogen configuration combinations and generates corresponding 3D structures derived from the supplied input conformers. For a fragment with N invertible nitrogen atoms this will lead to a multiplication of the number of fragment conformers by a factor of 2^N. In the current implementation a non-planar nitrogen atom is considered invertible if it has three single-bonded neighbors, is not a member of more than one ring and is connected to at least two heavy atoms. CONFORGE supports three nitrogen configuration enumeration modes: i) no enumeration - if specified, the enumeration procedure will be skipped and the fragment conformers are left unaltered, ii) only invertible nitrogens with undefined configuration are considered (default setting) and iii) all invertible nitrogens. Algorithmically, the configuration of a nitrogen is inverted by rotating the 3D coordinates of the atoms of a selected substituent to the exact opposite position on the other side of the plane spanned by the bonds to the remaining two substituents. If the inverted nitrogen atom is a ring member, the exocyclic substituent will be chosen for rotation. If the nitrogen is acyclic, the substituent comprising the smallest number of atoms is selected.

If the fragment contains rotatable acyclic bonds, each conformer in the current set will be subjected to torsion driving, further expanding the current fragment conformer ensemble by systematic sampling. Usually, torsion driving only needs to be carried out for flexible acyclic fragments for which just a single low-energy conformer is handed over by the parent

workflow (see supplementary section Fragment Conformer Ensemble Generation). Conceptually, the employed torsion driving workflow is largely similar to the one described for the generation of molecule conformers from a set of Fragment Conformer Combinations (FCC) (see main manuscript section Fragment Conformer Combination Torsion Driving) and therefore will not be outlined in more detail.

After torsion driving has finished, generated conformers which are out of the specified molecule conformer energy window get discarded and the remaining conformers are then forwarded to the last workflow step.

If no rotatable fragment bonds are present, the MMFF94 energy of each conformer using the force field parameterization of the parent molecule is calculated (the torsion driving procedure performs this step internally).

In the last post-processing step, the list of final fragment conformers is ordered by increasing energy and, if necessary, reduced to the maximum allowed output ensemble size (default setting: 10,000 conformers) by removing the necessary amount of high-energy conformers from the end of the list.

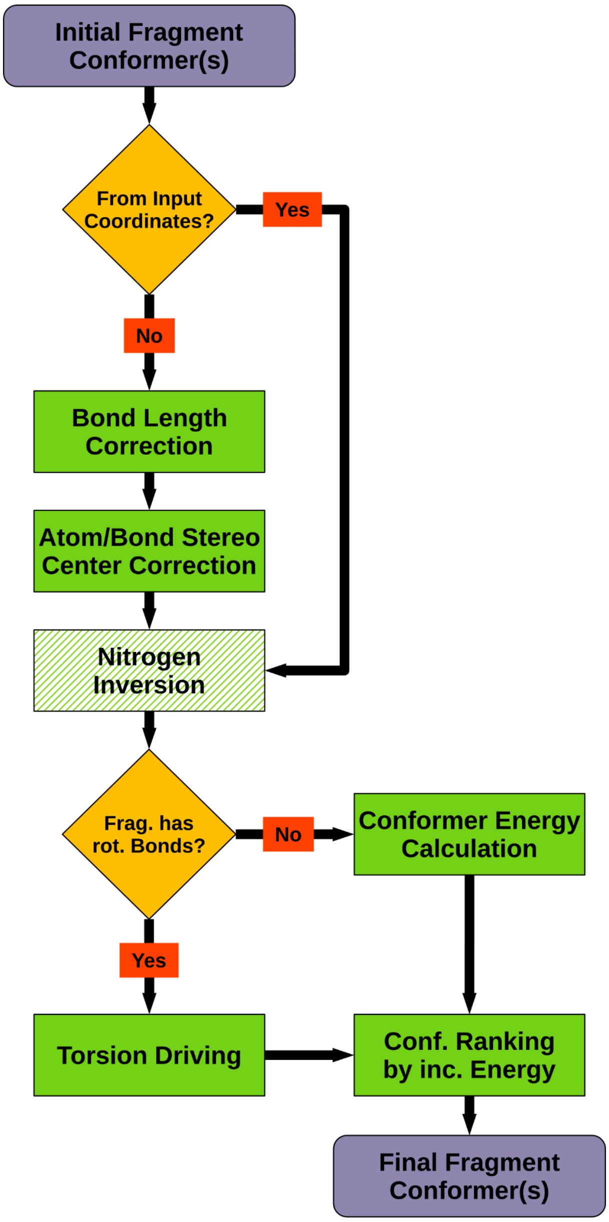

Figure S3. Fragment conformer post-processing workflow.

Multi-Molecule Output Conformer Ensemble Generation

For compounds that consist of multiple components an additional processing step is required which merges the separately generated molecule conformer ensembles into a single compound output conformer ensemble (see also main manuscript section Top-level Conformer Generation Workflow). The compound conformer generation method implemented in CONFORGE builds composite conformers from sets of selected molecule conformers by lining them up along the X-axis of the coordinate system. For each molecule conformer to be placed, an axis-aligned bounding box (AABB) is first calculated which specifies the minimum $[X_{min}, Y_{min}, Z_{min}]$ and maximum $[X_{max}, Y_{max}, Z_{max}]$ values of the atom coordinates in each dimension of space. The position $[X_{min}, (Y_{min} + Y_{max}) * 0.5, (Z_{min} + Z_{max}) * 0.5]$ then serves as an anchor point for calculating a translation vector to a particular location

on the X-axis. The placement X-position starts at *0* and gets incremented by X_{max} - X_{min} + *4.0* after each conformer has been placed. The constant *4.0* (in Å) represents an additional safety distance and makes sure that no atom Van der Waals sphere clashes occur between successively placed conformers. For the generation of the *i*th compound conformer, the molecule conformers at index *i* of the respective ensembles serve as input and its energy represents the total of the molecule conformer energies. If a conformer at index *i* does not exist, the molecule conformer with the highest index will be selected instead. Compound conformers are generated until all conformers of the largest molecule ensemble(s) have been consumed. A schematic representation of the compound conformer generation workflow can be found in Figure S4.

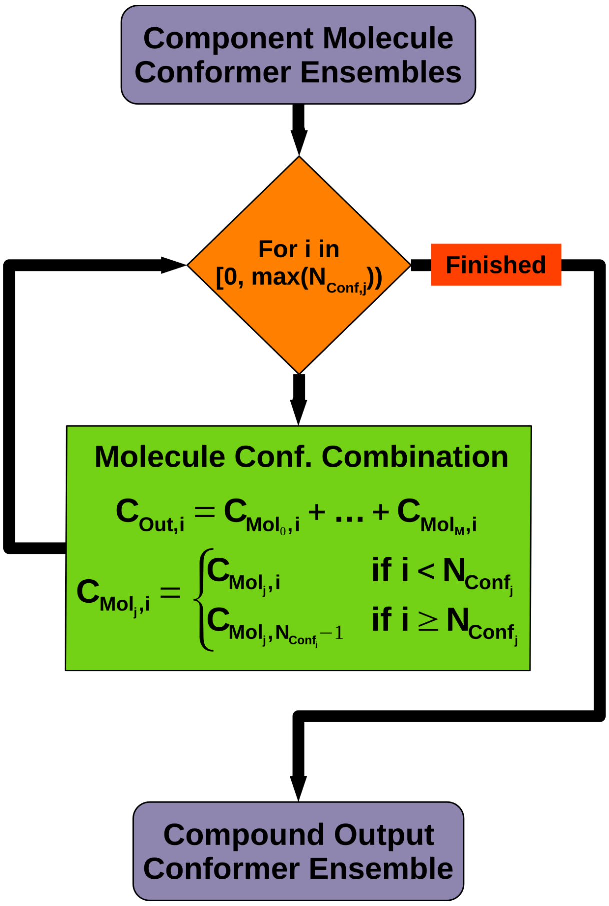

Figure S4. Output conformer ensemble generation workflow for multi-component compounds. $N_{Conf,j}/N_{Conf_j}$ = number of conformers of component *j*, $C_{Out,i}$ = *i*th compound output conformer, $C_{Mol_j,i}$ = *i*th conformer of component *j*, *M* = number of components.

Paired Wilcoxon Sign Rank Test[6] Results and Discussion

Platinum Diverse Dataset, maximum output ensemble size = 50

CONFORGE Systematic Best in comparison to other tested generators. CONFORGE Systematic Best is shown to produce a mean RMSD that is different to the one of other generators with high statistical significance ($\alpha = 0.05$, Table S1). As the two-sided p-Value is small and CONFORGE Systematic Best produces better results (more smaller RMSDs), which is in line with our prediction, we can use half of the p-Value, showing that CONFORGE Systematic Best produces better results than all other generators with high statistical significance.

Table S1. Paired Wilcoxon Sign Rank Test results for CONFORGE Systematic Best in comparison to other generators (Platinum Diverse Dataset with a max. output ensemble size of 50).

Generator	p-Value	Percentage RMSD ours smaller	Percentage RMSD theirs smaller	Percentage Ties
Balloon DG	<0.001	70.34%	29.66%	0.0%
Balloon GA	<0.001	73.95%	26.05%	0.0%
CONFORGE Systematic Default	<0.001	45.75%	26.69%	27.56%
Conformator Best	<0.001	54.29%	45.71%	0.0%
Conformator Fast	<0.001	64.34%	35.66%	0.0%
iCon Best	<0.001	60.72%	39.28%	0.0%
iCon Fast	<0.001	59.88%	40.12%	0.0%
RDKit ETKDGv3 UF	<0.001	57.35%	42.65%	0.0%
RDKit ETKDGv3 MF	<0.001	59.73%	40.27%	0.0%

CONFORGE Systematic Default in comparison to other tested generators. CONFORGE Systematic Default is shown to produce a mean RMSD that is different to the one of other generators with high statistical significance ($\alpha = 0.05$, Table S2), except for Conformator Best, for which we cannot reject the null hypothesis that the RMSD results follow the same random distribution, and CONFORGE Systematic Best which is better than CONFORGE Systematic Default with high statistical significance. For the other generators, as the two-sided p-Value is small and CONFORGE Systematic Default produces better results (more smaller RMSDs), which is in line with our prediction, we can use half of the p-Value, showing that CONFORGE Systematic Default produces a better result with high statistical significance.

Table S2. Paired Wilcoxon Sign Rank Test results for CONFORGE Systematic Default in comparison to other generators (Platinum Diverse Dataset with a max. output ensemble size of 50).

Generator	p-Value	Percentage RMSD ours smaller	Percentage RMSD theirs smaller	Percentage Ties
Balloon DG	<0.001	65.37%	34.63%	0.0%
Balloon GA	<0.001	70.55%	29.45%	0.0%
CONFORGE Systematic Best	<0.001	26.69%	45.75%	27.56%
Conformator Best	1.0	48.93%	51.07%	0.0%
Conformator Fast	<0.001	58.49%	41.51%	0.0%
iCon Best	<0.001	55.47%	44.53%	0.0%
iCon Fast	0.003	51.8%	48.2%	0.0%
RDKit ETKDGv3 UF	<0.001	52.59%	47.41%	0.0%
RDKit ETKDGv3 MF	<0.001	55.46%	44.54%	0.0%

Platinum Diverse Dataset, maximum output ensemble size = 250

CONFORGE Systematic Best in comparison to other tested generators. CONFORGE Systematic Best is shown to produce a mean RMSD that is different to the one of other generators with high statistical significance ($\alpha = 0.05$, Table S3). As the two-sided p-Value is small and CONFORGE Systematic Best produces better results (more smaller RMSDs), which is in line with our prediction, we can use half of the p-Value, showing that CONFORGE Systematic Best produces better results than all other generators with high statistical significance.

Table S3. Paired Wilcoxon Sign Rank Test results for CONFORGE Systematic Best in comparison to other generators (Platinum Diverse Dataset with a max. output ensemble size of 250).

Generator	p-Value	Percentage RMSD ours smaller	Percentage RMSD theirs smaller	Percentage Ties
Balloon DG	<0.001	65.57%	34.43%	0.0%
Balloon GA	<0.001	70.9%	29.1%	0.0%
CONFORGE Systematic Default	<0.001	52.96%	19.62%	27.42%
Conformator Best	0.029	52.19%	47.81%	0.0%
Conformator Fast	<0.001	65.74%	34.26%	0.0%
iCon Best	<0.001	66.95%	33.05%	0.0%
iCon Fast	<0.001	59.67%	40.33%	0.0%
RDKit ETKDGv3 UF	<0.001	59.94%	40.06%	0.0%
RDKit ETKDGv3 MF	<0.001	60.57%	39.43%	0.0%

CONFORGE Systematic Default in comparison to other tested generators. CONFORGE Systematic Default is shown to produce a mean RMSD that is different to the one of other generators with high statistical significance ($\alpha = 0.05$, Table S4), except for iCon Fast, RDKit ETKDGv3 UF and RDKit ETKDGv3 MF, for which we cannot reject the null hypothesis that the RMSD results follow the same random distribution. For the generators Balloon DG, Balloon GA, Conformator Fast and iCon Best, as the two-sided p-Value is small and CONFORGE Systematic Default produces better results (more smaller RMSDs), which is in line with our prediction, we can use half of the p-Value, showing that CONFORGE Systematic Default produces a better result with high statistical significance. For the remaining generators, namely CONFORGE Systematic Best and Conformator Best, we cannot claim RMSD improvements as statistically significant differences have been shown and our prediction of better average RMSD was not shown to be true.

Table S4. Paired Wilcoxon Sign Rank Test results for CONFORGE Systematic Default in comparison to other generators (Platinum Diverse Dataset with a max. output ensemble size of 250).

Generator	p-Value	Percentage RMSD ours smaller	Percentage RMSD theirs smaller	Percentage Ties
Balloon DG	<0.001	57.49%	42.51%	0.0%
Balloon GA	<0.001	63.06%	36.94%	0.0%
CONFORGE Systematic Best	<0.001	19.62%	52.96%	27.42%
Conformator Best	<0.001	41.89%	58.11%	0.0%
Conformator Fast	<0.001	54.29%	45.71%	0.0%
iCon Best	<0.001	55.93%	44.07%	0.0%
iCon Fast	0.164	47.04%	52.96%	0.0%
RDKit ETKDGv3 UF	1.0	48.85%	51.15%	0.0%
RDKit ETKDGv3 MF	0.732	49.44%	50.56%	0.0%

Prime Macrocycle Dataset, maximum output ensemble size = 500

CONFORGE Stochastic in comparison to other tested generators. CONFORGE Stochastic is shown to produce a mean RMSD that is different to the one of other generators with high statistical significance ($\alpha = 0.05$, Table S5), except for RDKit ETKDGv3 MF for which we cannot reject the null hypothesis that the RMSD results follow the same random distribution. For the other generators, as the two-sided p-Value is small and CONFORGE Stochastic produces better results (more smaller RMSDs), which is in line with our prediction, we can use half of the p-Value, showing that CONFORGE Stochastic produces a better result with high statistical significance.

Table S5. Paired Wilcoxon Sign Rank Test results for CONFORGE Stochastic in comparison to other generators (Prime Dataset with a max. output ensemble size of 500).

Generator	p-Value	Percentage RMSD ours smaller	Percentage RMSD theirs smaller	Percentage Ties
Balloon DG	<0.001	86.43%	13.57%	0.0%
Balloon GA	<0.001	96.95%	3.05%	0.0%
Conformator Best	<0.001	81.09%	18.91%	0.0%
Conformator Fast	<0.001	86.93%	13.07%	0.0%
iCon Best	<0.001	78.74%	21.26%	0.0%
iCon Fast	<0.001	73.43%	26.57%	0.0%
RDKit ETKDGv3 UF	<0.001	67.79%	32.21%	0.0%
RDKit ETKDGv3 MF	1.0	50.48%	49.52%	0.0%

Table S6. CONFORGE Command-line Interface Options

Short [Long] Option	Default Argument	Description	Default Setting
**Mandatory Options**			
-i [--input] arg(s)	-	Input file(s).	-
-o [--output] arg	-	Output file.	-
**Miscellaneous Options**			
-h [--help] (arg)	SHORT	Print help message and exit (values: ABOUT, USAGE, SHORT, ALL or 'name of option').	-
-V [--version]	-	Print version information and exit.	-
-v [--verbosity] (arg)	VERBOSE	Verbosity level of information output (values: QUIET, ERROR, INFO, VERBOSE, DEBUG).	INFO
-c [--config] arg	-	Use file with program options.	-
-l [--log-file] arg	-	Redirect text-output to file.	-
-p [--progress] (arg)	true	Show progress bar.	true
-t [--num-threads] (arg)	Number of detected CPU-cores	Number of parallel execution threads (value must be >= 0; 0 disables multithreading).	0
**I/O Options**			
-I [--input-format] arg	-	Input file format.	auto-detect from file-ext.
-O [--output-format] arg	-	Output file format.	auto-detect from file-ext.
-F [--failed-format] arg	-	Failed molecule output file format.	auto-detect from file-ext.
-Y [--energy-sd-entry] (arg)	true	Output conformer energy in the structure data section of SD-files.	false
-M [--energy-comment] (arg)	true	Output conformer energy in the comment field (only effective if supported by output format).	false
-W [--conf-idx-suffix] (arg)	true	Append conformer index to the title of multi-conf. output molecules.	false
**Options controlling Conformer Generation**			
-C [--conf-gen-preset] arg	-	Conformer generation preset to use (values: SMALL_SET_DIVERSE, MEDIUM_SET_DIVERSE, LARGE_SET_DIVERSE, SMALL_SET_DENSE, MEDIUM_SET_DENSE, LARGE_SET_DENSE).	MEDIUM_SET_DIVERSE
-f [--failed] arg	-	Failed molecule output file.	-

-m [--mode] arg	-	Conformer sampling mode (values: AUTO, STOCHASTIC, SYSTEMATIC).	AUTO
-e [--e-window] arg	-	Output energy window for generated conformers (must be >= 0).	15 kcal/mol
-r [--rmsd] arg	-	Minimum RMSD for output conformer selection (must be >= 0; 0 disables RMSD checking)	0.5 Å
-n [--max-num-out-confs] arg	-	Maximum number of output conformers per molecule (must be >= 0; 0 disables limit).	100
-N [--nitrogen-enum-mode] arg	-	Invertible nitrogen enumeration mode (values: NONE, ALL, UNSPECIFIED).	UNSPECIFIED
-R [--enum-rings] (arg)	true	Enumerate ring conformers (only effective in systematic sampling mode).	true
-H [--sample-het-hydrogens] (arg)	true	Perform torsion sampling for hydrogens on hetero atoms.	false
-A [--tol-range-sampling] (arg)	true	Additionally generate conformers for angles at the boundaries of the first torsion angle tolerance range (only effective in systematic sampling mode).	false
-u [--include-input] (arg)	true	Add input 3D-structure to output conformer ensemble.	false
-S [--from-scratch] (arg)	true	Discard input 3D-coordinates and generate conformers from scratch.	true
-d [--systematic-search-force-field] arg	-	Search force field used for systematic sampling (values: MMFF94, MMFF94_NO_ESTAT, MMFF94S, MMFF94S_XOOP, MMFF94S_RTOR, MMFF94S_RTOR_XOOP, MMFF94S_NO_ESTAT, MMFF94S_XOOP_NO_ESTAT, MMFF94S_RTOR_NO_ESTAT, MMFF94S_RTOR_XOOP_NO_ESTAT).	MMFF94S_RTOR_NO_ESTAT
-q [--stochastic-search-force-field] arg	-	Search force field used for stochastic sampling (values: MMFF94, MMFF94_NO_ESTAT, MMFF94S, MMFF94S_XOOP, MMFF94S_RTOR, MMFF94S_RTOR_XOOP, MMFF94S_NO_ESTAT, MMFF94S_XOOP_NO_ESTAT, MMFF94S_RTOR_NO_ESTAT, MMFF94S_RTOR_XOOP_NO_ESTAT).	MMFF94S_RTOR
-s [--strict-param] (arg)	true	Perform strict MMFF94 parameterization.	true
-D [--dielectric-const] arg	-	Dielectric constant used for the calculation of electrostatic interaction energies.	80
-E [--dist-exponent] arg	-	Distance exponent used for the calculation of electrostatic interaction energies.	1

-T [--timeout] arg	-	Time in seconds after which molecule conformer generation will be stopped (value must be >= 0; 0 disables timeout).	3600 s
-X [--max-num-rot-bonds] arg	-	Maximum number of allowed rotatable bonds, exceeding this limit causes molecule conf. generation to fail (negative values disable limit).	-1
-L [--max-pool-size] arg	-	Puts an upper limit on the number of generated output conformer candidates (only effective in systematic sampling mode; value must be >= 0; 0 disables limit).	10000
-x [--max-num-sampled-confs] arg	-	Maximum number of sampled conformers (only effective in stochastic sampling mode; value must be >= 0; 0 disables limit).	2000
-y [--conv-check-cycle-size] arg	-	Minimum number of duplicate conformers that have to be generated in succession to consider convergence to be reached (only effective in stochastic sampling mode; value must be > 0).	100
-Z [--mc-rot-bond-count-thresh] arg	-	Number of rotatable bonds in a ring above which stochastic sampling will be performed (only effective in sampling mode AUTO; value must be > 0).	10
-P [--ref-tol] arg	-	Energy tolerance at which force field structure refinement stops (only effective in stochastic sampling mode; value must be >= 0; 0 results in refinement until convergence).	0.001 kcal/mol
-w [--max-ref-iter] arg	-	Maximum number of force field structure refinement iterations (only effective in stochastic sampling mode; value must be >= 0; 0 disables limit).	0
-k [--add-tor-lib] arg	-	Torsion library to be used in addition to the built-in library (only effective in systematic sampling mode).	-
-K [--set-tor-lib] arg	-	Torsion library used as a replacement for the built-in library (only effective in systematic sampling mode).	-
-B [--frag-build-preset] arg	-	Fragment build preset to use (values: FAST, THOROUGH; only effective in systematic sampling mode).	FAST
-b [--build-force-field] arg	-	Fragment build force field (values: MMFF94, MMFF94_NO_ESTAT, MMFF94S, MMFF94S_XOOP, MMFF94S_RTOR, MMFF94S_RTOR_XOOP, MMFF94S_NO_ESTAT, MMFF94S_XOOP_NO_ESTAT, MMFF94S_RTOR_NO_ESTAT, MMFF94S_RTOR_XOOP_NO_ESTAT; only effective in systematic sampling mode).	MMFF94S_RTOR_NO_ESTAT
-g [--add-frag-lib] arg	-	Fragment library to be used in addition to the built-in library (only effective in systematic sampling mode).	-
-G [--set-frag-lib] arg	-	Fragment library used as a replacement for the built-in library (only effective in systematic sampling mode).	-

-z [--canonicalize] (arg)	true	Canonicalize input molecules.	false

References

(1) McKay, B. Practical Graph Isomorphism. In *Numerical mathematics and computing*; 1981.

(2) Eastlake, D.; Jones, P. RFC3174: US Secure Hash Algorithm 1 (SHA1), Internet Engineering Task Force. 2001.

(3) Blaney, J. M.; Dixon, J. S. Distance Geometry in Molecular Modeling. In *Reviews in Computational Chemistry*; John Wiley & Sons, Inc.: Hoboken, NJ, USA, 2007; pp 299–335. https://doi.org/10.1002/9780470125823.ch6.

(4) Crippen, G. M.; Havel, T. F. Distance Geometry and Molecular Conformation. **1988**.

(5) Halgren, T. A. Merck Molecular Force Field. I. Basis, Form, Scope, Parameterization, and Performance of MMFF94. *J. Comput. Chem.* **1996**, *17* (5-6), 490–519. https://doi.org/10.1002/(SICI)1096-987X(199604)17:5/6<490::AID-JCC1>3.0.CO;2-P.

(6) Blair, R. C.; Higgins, J. J. Comparison of the Power of the Paired Samples T Test to that of Wilcoxon's Signed-Ranks Test under Various Population Shapes. *Psychol. Bull.* **1985**, *97* (1), 119.
